# The GATA Transcription Factor *egl-27* Delays Aging by Promoting Stress Resistance in *Caenorhabditis elegans*

**DOI:** 10.1371/journal.pgen.1003108

**Published:** 2012-12-13

**Authors:** Xiao Xu, Stuart K. Kim

**Affiliations:** Cancer Biology Program and Departments of Developmental Biology and Genetics, Stanford University School of Medicine, Stanford, California, United States of America; The University of North Carolina at Chapel Hill, United States of America

## Abstract

Stress is a fundamental aspect of aging, as accumulated damage from a lifetime of stress can limit lifespan and protective responses to stress can extend lifespan. In this study, we identify a conserved *Caenorhabditis elegans* GATA transcription factor, *egl-27*, that is involved in several stress responses and aging. We found that overexpression of *egl-27* extends the lifespan of wild-type animals. Furthermore, *egl-27* is required for the pro-longevity effects from impaired insulin/IGF-1 like signaling (IIS), as reduced *egl-27* activity fully suppresses the longevity of worms that are mutant for the IIS receptor, *daf-2*. *egl-27* expression is inhibited by *daf-2* and activated by pro-longevity factors *daf-16*/FOXO and *elt-3*/GATA, suggesting that *egl-27* acts at the intersection of IIS and GATA pathways to extend lifespan. Consistent with its role in IIS signaling, we found that *egl-27* is involved in stress response pathways. *egl-27* expression is induced in the presence of multiple stresses, its targets are significantly enriched for many types of stress genes, and altering levels of *egl-27* itself affects survival to heat and oxidative stress. Finally, we found that *egl-27* expression increases between young and old animals, suggesting that increased levels of *egl-27* in aged animals may act to promote stress resistance. These results identify *egl-27* as a novel factor that links stress and aging pathways.

## Author Summary

Stress is a fundamental aspect of aging, but it is unclear whether the molecular mechanisms underlying stress response become altered during normal aging and whether these alterations can affect the aging process. In this study, we found a GATA transcription factor called *egl-27*, whose targets are significantly enriched for age-dependent genes and stress response genes, and whose expression increases with age. In contrast to previous work describing factors that are causal for aging, we found that *egl-27* activity is likely beneficial for survival since *egl-27* overexpression extends lifespan. *egl-27* promotes longevity by enhancing stress response; specifically, increased levels of *egl-27* protect animals against heat stress, while reduced *egl-27* activity impairs survival following heat and oxidative stress. These results suggest that aging is not simply a process of constant decline. Some factors, such as *egl-27*, are more active in old animals, working to restore organismal function and to improve survival.

## Introduction

Responses to various forms of stress play an important role in aging and longevity. Several types of stress result in damage that can accumulate over time (e.g. oxidative stress results in damaged proteins that often accumulate with age) [1]–[3]. Responses to these stresses have protective effects that can alleviate the effects of damage accumulation. Consistent with this idea, previous studies have found that mutants with extended longevity often exhibit increased stress resistance [4]–[7]. For example, mutations that disrupt the insulin/IGF-1 like signaling (IIS) pathway not only extend longevity, but also increase resistance to many types of stress including heat, oxidative, and pathogenic stress [8]–[11].

As the first genetic pathway in *Caenorhabditis elegans* that was linked to longevity, the IIS pathway is a conserved endocrine component that controls important aspects of development, metabolism, and stress response [12]. Activation of the IIS receptor (DAF-2) causes phosphorylation of a phosphatidylinositol 3-kinase (AGE-1), which initiates a cascade of signals resulting in phosphorylation and inactivation of a FOXO transcription factor (DAF-16). Reduction of IIS through knockdown of *daf-2* or through the presence of certain environmental stresses, results in activation of DAF-16/FOXO, which triggers a transcriptional program that promotes both stress resistance and longevity [10], [12]. Many genes in the DAF-16 transcriptional response are involved in various stress responses, and some of these also change in expression during aging. For example, heat shock proteins are induced by many types of stress including heat and pathogenic infection [13]–[17], and expression levels of certain heat shock genes are increased in *C. elegans* mutants with reduced IIS and extended longevity. Furthermore, heat shock proteins in *C. elegans* increase in expression between young and old animals, although expression is reduced in very old populations in which 90% of the population is dead [18], [19].

The increased expression of stress genes during aging is not confined to worms. Studies have shown that genes induced by oxidative stress increase with age in flies [20]; p53-related damage response genes increase with age in mice [21]; also, genes that are involved in immunological complement activation, which are generally induced in response to oxidative and pathogenic stress [22]–[26], increase expression in old age across four human tissues [27]. While these results suggest that stress response pathways become increasingly activated in old organisms, it is unclear whether this activation has a protective function and is beneficial for longevity or whether it represents a misregulation of stress pathways and is a contributor to organismal decline.

In *C. elegans*, the upstream regions of the genes that constitute the DAF-16 transcriptional program are enriched for both the DAF-16 binding site and a GATA-like transcription factor binding site [28]. One of the GATA factors that may be involved in the DAF-16 mediated IIS transcriptional program is ELT-3, as *elt-3* expression is increased in *age-1* mutants. Furthermore, *elt-3* is required for the longevity phenotype of *daf-2* mutants, suggesting that the *elt-3*/GATA transcription factor functions downstream of the IIS pathway [29].

The GATA family of transcription factors may also play important roles in regulating the molecular changes that accompany normal aging. Transcriptional profiling of young and old animals has revealed that the promoters of age-dependent genes are enriched for GATA motifs. The GATA transcription factor *elt-3* is responsible for some of the age-dependent changes in gene expression. Expression of *elt-3* declines as worms age, resulting in decreased expression of its downstream targets. Low levels of *elt-3* have a deleterious effect on survival and stress response suggesting that this decline in *elt-3* levels hastens the aging process [29].

In this work, we identify another GATA transcription factor, *egl-27*, that functions to promote stress survival and to delay aging. In addition to its homology to GATA factors, *egl-27* is also homologous to the MTA1 component of NuRD chromatin remodeling complex [30]–[33]. Previous studies show that *egl-27* is expressed in most somatic cells during development and in adult worms [30], [34]. We show that *egl-27* expression increases with age and that increased levels of *egl-27* through overexpression are sufficient to extend lifespan and to increase survival in response to heat stress. In contrast, reducing *egl-27* activity suppresses the longevity and thermotolerance phenotypes of reduced insulin/IGF-1 like signaling. Moreover, *egl-27* can respond to the presence of stress as its expression is induced by a variety of different stresses. EGL-27 binds upstream of genes involved in both stress and aging, but interestingly EGL-27 targets are enriched for genes whose expression decreases with age. Finally, *egl-27* expression is regulated by the GATA transcription factor *elt-3* and the IIS transducing gene *daf-16.* These results define *egl-27* as a novel factor that promotes both longevity and stress response.

## Results

### *egl-27* functions to promote longevity

**Figure 1:**
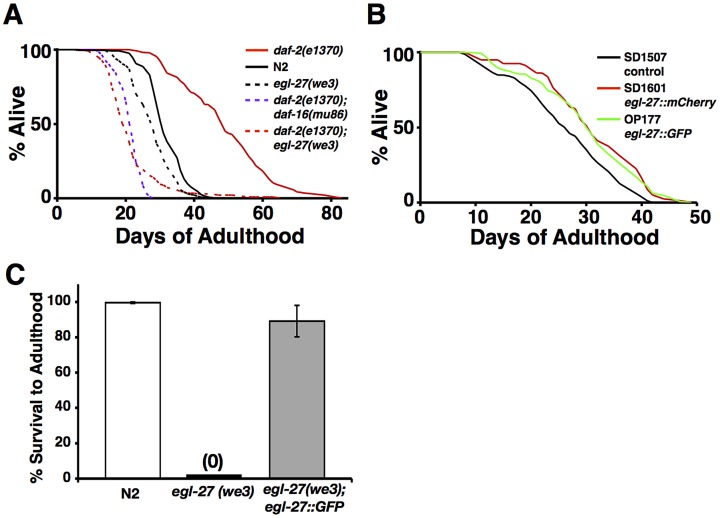
*egl-27* functions to promote longevity. (A) *egl-27(we3)* completely suppresses the longevity phenotype of *daf-2(e1370)* mutants. All worms were hatched at 20°C and shifted to 15°C at day 2 of adulthood. *daf-2(e1370)*: n = 279, mean lifespan = 48.9 days, median lifespan = 49 days, 95% mortality = 70 days. *daf-2(e1370); daf-16(mu86)*: n = 209, mean lifespan = 24.0 days, median lifespan = 25 days, 95% mortality = 30 days. *daf-2(e1370); egl-27(we3)*: n = 234, mean lifespan = 21.5 days, median lifespan = 20 days, 95% mortality = 36 days. p = 0; log rank test comparing *daf-2(e1370); egl-27(we3)* vs. *daf-2(e1370).* N2: n = 246, mean lifespan = 31.6 days, median lifespan = 31 days, 95% mortality = 41 days. *egl-27(we3)*: n = 232, mean lifespan = 27.8 days, median lifespan = 28 days, 95% mortality = 37 days. p = 7.8×10^−10^ vs. N2. Additional lifespan data can be found in Table S1. (B) OP177 and SD1601, two independent strains overexpressing *egl-27* extend lifespan compared to SD1507, transgenic control worms. SD1507 (control): n = 165, mean lifespan = 26.3 days, median lifespan = 27 days, 95% mortality = 40 days. SD1601 (*egl-27::mCherry*): n = 80, mean lifespan = 31 days, median lifespan = 31 days, 95% mortality = 42 days. p-value = 4.0×10^−5^ vs. control. OP177 (*egl-27::GFP*): n = 146, mean lifespan = 30.1 days, median lifespan = 30.5 days, 95% mortality = 44 days. p-value = 4.9×10^−5^ vs. control. Additional lifespan data can be found in Table S1. (C) *egl::GFP* rescues the larval arrest and embryonic lethality phenotypes of *egl-27(we3)* mutants. Error bars indicate SEM across 10 independent experiments. Total number of eggs across all 10 experiments for N2 = 167, *egl-27(we3)* = 154, *egl-27(we3); egl-27::GFP* = 164.

Reduction of *egl-27* activity by RNAi knockdown was previously shown to partially suppress the longevity phenotype of the long-lived IIS receptor mutant *daf-2(e1370)* [29]. We extended this result by showing that the cold-sensitive *egl-27(we3)* allele could fully suppress the longevity phenotype of *daf-2(e1370)*. Specifically, we found that *daf-2(e1370); egl-27(we3)* double mutants have a median lifespan that is 2.5 fold shorter than *daf-2(e1370)* single mutants and 35% shorter than wild-type worms (Figure 1A). *egl-27(we3)* mutants live only 10% shorter than wild-type worms, suggesting that the combination of the *egl-27(we3)* mutation and the *daf-2(e1370)* mutation results in a slight synthetic lethality (Figure 1A).

As a control, we compared the extent of *daf-2(e1370)* suppression by *egl-27(we3)* to that of *daf-16*/FOXO, a well-characterized suppressor of *daf-2* [35]. We found that *daf-2(e1370); daf-16(mu86)* double mutants have a median lifespan that is two-fold shorter than *daf-2(e1370)* single mutants and 19% shorter than wild-type worms (Figure 1A). These data show that *egl-27(we3)* suppresses *daf-2(e1370)* longevity to approximately the same extent as *daf-16(mu86)*.

*egl-27(we3)* is cold-sensitive for lethality [30], and so we tested whether temperature affects the suppression of *daf-2* longevity by *egl-27(we3)*. We hatched worms at the developmentally permissive temperature (20°C) and then shifted them at day 2 of adulthood to 15°C, 20°C, or 25°C. We found that *egl-27(we3)* suppresses *daf-2(e1370)* longevity at all three temperatures; specifically, *daf-2(e1370); egl-27(we3)* worms have a median lifespan that is 2.9 fold shorter than *daf-2(e1370)* mutants at 15°C, 1.9 fold shorter at 20°C, and 1.7 fold shorter at 25°C (Figure S1A). These results show that *egl-27(we3)* is temperature-sensitive for developmental arrest but not for suppression of longevity by *daf-2(e1370)*.

We next tested whether increased levels of *egl-27* are sufficient to increase longevity. To do this, we engineered three different constructs containing *egl-27* and generated strains overexpressing each construct. The first construct is from the modENCODE project and contains GFP-tagged *egl-27* in a fosmid with 18 kb of sequence upstream and 8 kb of sequence downstream of *egl-27*. This fosmid also contains the full coding sequence for three other genes: *F31E8.6*, *F31E8.1*, and *tbc-1*. We found that worms expressing *egl-27::GFP* had a lifespan extension of 13% (Figure 1B). The second construct contains mCherry-tagged *egl-27* with full intergenic regions covering 5 kb of sequence upstream and 152 bp of sequence downstream of *egl-27*. Worms overexpressing *egl-27::mCherry* had a lifespan extension of 15% (Figure 1B). Finally, we cloned the *egl-27* genomic region containing the *we3* temperature-sensitive mutation from *egl-27(we3)* worms. This construct also contains full intergenic regions. We generated three transgenic worm strains containing *egl-27(we3)* on an extrachromosomal array in order to create strains that conditionally overexpress *egl-27* at the permissive temperature. We grew worms at either the non-permissive or permissive temperature starting at day two of adulthood, and then measured their lifespans. Interestingly, we found that overexpression of *egl-27(we3)* extended lifespan at both the permissive and non-permissive temperatures. Specifically, at 20°C, median lifespan was increased 23–31%(Figure S1B) and at 15°C, the median lifespan was increased 11–21% (Figure S1C). These results suggest that the addition of low levels of *egl-27(we3)* activity at 15°C is sufficient to extend lifespan or that *egl-27(we3)* is temperature sensitive for development but not for its life-extending functions.

To determine whether *egl-27* is expressed at higher levels in these overexpression lines compared to control worms, we used qRT-PCR to measure levels of *egl-27* mRNA expression in the different overexpression strains and in control worms. We found that *egl-27* expression is increased 2.4 fold in the *egl-27::GFP* strain versus control worms (p = 0.0008, Figure S5E). *egl-27* expression is increased 4.1 fold in the *egl-27::mCherry* strain (p = 0.02, Figure S1D). Finally, *egl-27* expression is increased 23%, 11%, and 2.4 fold in the three *egl-27(we3)* overexpression strains although none of these increases are significant, possibly due to high expression variability caused by extrachromosomal array expression (Figure S1D). We did not observe any abnormal developmental phenotypes in any of the *egl-27* overexpression lines, suggesting that these levels of increased *egl-27* do not adversely affect development. Furthermore, we validated that transgenic *egl-27::GFP* can function like endogenous *egl-27(+)*, as we showed that *egl-27::GFP* can rescue the *egl-27(we3)* lethal mutant phenotype (Figure 1C).

### *egl-27* functions to promote heat and oxidative stress survival

Increased longevity is strongly correlated with increased stress resistance [4], [36]. To determine whether *egl-27* can promote stress survival, we assessed the relationship between *egl-27* activity and survival to heat and oxidative stress. To assay the phenotype of an *egl-27* reduction-of-function mutation, we used the hypomorphic mutation *egl-27(we3)*. To assay the phenotype from overexpression of *egl-27*, we used the *egl-27::GFP* strain described above.

**Figure 2:**
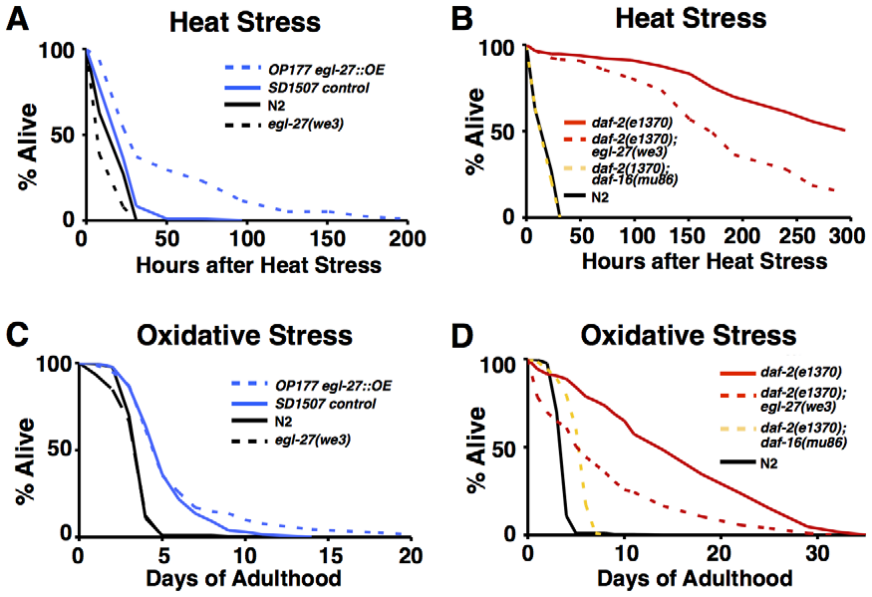
*egl-27(+)* promotes heat and oxidative stress survival. For A and B, day 1 adult hermaphrodite worms worms were heat stressed at 35°C for 8 hours and survival was scored at 20°C as a function of time. Additional survival data can be found in Table S1. (A) *egl-27(we3)* worms have increased sensitivity to heat-stress while *egl-27::GFP* worms are more resistant to heat stress. N2: n = 129, mean survival = 19.6 hours, median survival = 23 hours, 95% mortality = 31 hours. *egl-27(we3)*: n = 114, mean survival = 14.4 hours, median survival = 9 hours, 95% mortality = 31 hours. p = 7.8×10^−8^ vs. N2. Control: n = 108, mean survival = 24.6 hours, median survival = 23 hours, 95% mortality = 50 hours. *egl-27::GFP*: n = 78, mean survival = 52.4 hours, median survival = 31 hours, 95% mortality = 134 hours. p = 5.8×10^−6^ vs. control. (B) Loss of *egl-27* activity partially suppresses the stress resistance of *daf-2(e1370)* mutants. *daf-2(e1370)* : n = 113, mean survival = 238 hours, median survival = 296 hours. *daf-2(e1370)*; *egl-27(we3)*: n = 118, mean survival = 183 hours, median survival = 172 hours. p = 7.8×10^−10^ vs. *daf-2(e1370*). *daf-2(e1370)*; *daf-16(mu86)*: n = 128, mean survival = 19.2 hours, median survival = 23 hours, 95% mortality = 31 hours. N2: n = 129, mean survival = 19.6 hours, median survival = 23 hours, 95% mortality = 31 hours. For C and D, day 1 adult hermaphrodite worms were grown on plates supplemented with 10 mM paraquat and 30 mM FuDR and survival was scored at 20°C as a function of time. Additional survival data can be found in Table S1. (C) *egl-27* does not affect paraquat survival in wild-type and control worms. N2: n = 212, mean survival = 3.8 days, median survival = 4 days, 95% mortality = 5 days. *egl-27(we3)*: n = 243, mean survival = 3.6 days, median survival = 4 days, 95% mortality = 5 days. Control: n = 133, mean survival = 5.4 days, median survival = 5 days, 95% mortality = 9 days. *egl-27::GFP*: n = 272, mean survival = 6.1 days, median survival = 5 days, 95% mortality = 14 days. (D) *daf-2(e1370); egl-27(we3)* double mutants are less resistant to paraquat-induced oxidative stress than *daf-2(e1370)* mutants. *daf-2(e1370)*: n = 211, mean survival = 15.7 days, median survival = 14 days, 95% mortality = 29 days. *daf-2(e1370)*; *egl-27(we3)*: n = 284, mean survival = 8.2 days, median survival = 6 days, 95% mortality = 25 days (p-value vs. *daf-2(e1370)* = 0). *daf-2(e1370)*; *daf-16(mu86)*: n = 262, mean survival = 5.4 days, median = 6 days, 95% mortality = 7 days. N2: n = 212, mean survival = 3.8 days, median survival = 4 days, 95% mortality = 5 days.

We assayed heat-stress survival by subjecting worms to 8 hours at 35°C and then measuring survival (Figure 2A). *egl-27* reduction-of-function mutants die more quickly after heat stress than wild-type worms; *egl-27(we3)* has a median survival time following heat-stress that is 2.9 fold shorter than for wild-type worms (log rank p-value = 7.8×10^−8^). *egl-27* gain-of-function worms survive longer after heat stress than control worms; they have a median survival time following heat stress that is 1.4 fold longer than for control worms (p = 5.8×10^−6^) and they also have a time to 95% mortality that is 2.6 fold longer than for control worms.

*daf-2* insulin-like receptor mutants are resistant to many types of stress [9], [11]. We showed that *egl-27(we3)* partially suppresses the heat resistance phenotype of *daf-2*. *daf-2(e1370); egl-27(we3)* double mutants have a median survival that is 1.7 fold shorter than *daf-2(e1370)* single mutants, but still 7.5 fold longer than wild-type worms. In contrast, a well-characterized suppressor of *daf-2*, the FOXO transcription factor *daf-16(mu86)* fully suppresses the heat stress resistance conferred by *daf-2* mutants. *daf-2(e1370); daf-16(mu86)* double mutants have a median survival that is 12.9 fold shorter than *daf-2(e1370)* single mutants and that is the same as wild-type worms (Figure 2B). Additionally, *egl-27(we3)* has a less pronounced effect on the heat stress survival of *daf-2(e1370)* mutants than it does on wild-type worms (1.7 fold shorter vs. 2.9 fold shorter respectively) suggesting that *egl-27* may be required for some but not all of the heat-stress resistance of *daf-2(e1370)* mutants.

We examined whether *egl-27* has a functional role in mediating the response to oxidative stress. To do this, we grew worms on plates supplemented with 10 mM paraquat and then measured their survival time. We observed that altering levels of *egl-27* did not have a large effect on oxidative stress survival as neither reduction nor gain of *egl-27* activity affects median lifespan compared to control worms (Figure 2C). However, we found that *egl-27(we3)* partially suppresses the oxidative stress resistant phenotype of *daf-2(e1370)*; specifically, *daf-2(e1370); egl-27(we3)* double mutants have a median lifespan that is 2.5-fold shorter than for *daf-2(e1370)* mutants and that is 1.4-fold longer than wild-type worms (Figure 2D). Although the median survival time for *daf-2(e1370); egl-27(we3)* double mutants is similar to that of *daf-2(e1370); daf-16(mu86)* double mutants (which have a median time of survival that is 2.3-fold shorter than for *daf-2(e1370)* mutants and that is 1.5-fold longer than for wild-type worms), the time to 95% mortality is different between the two lines. While *daf-2(e1370); daf-16(mu86)* double mutants have a time to 95% mortality that is 4.1 fold shorter than for *daf-2(e1370)* single mutants, *daf-2(e1370); egl-27(we3)* double mutants have a time to 95% mortality that is 16% shorter than for *daf-2(e1370)* single mutants (Figure 2D). These results suggest that *egl-27* activity is important for some of the oxidative stress resistance conferred by reduced activity of the insulin-like receptor gene *daf-2*.

### *egl-27* acts downstream of *daf-2*/ILR and *daf-16*/FOXO in the IIS pathway, and downstream of the *elt-3*/GATA transcription factor

**Figure 3:**
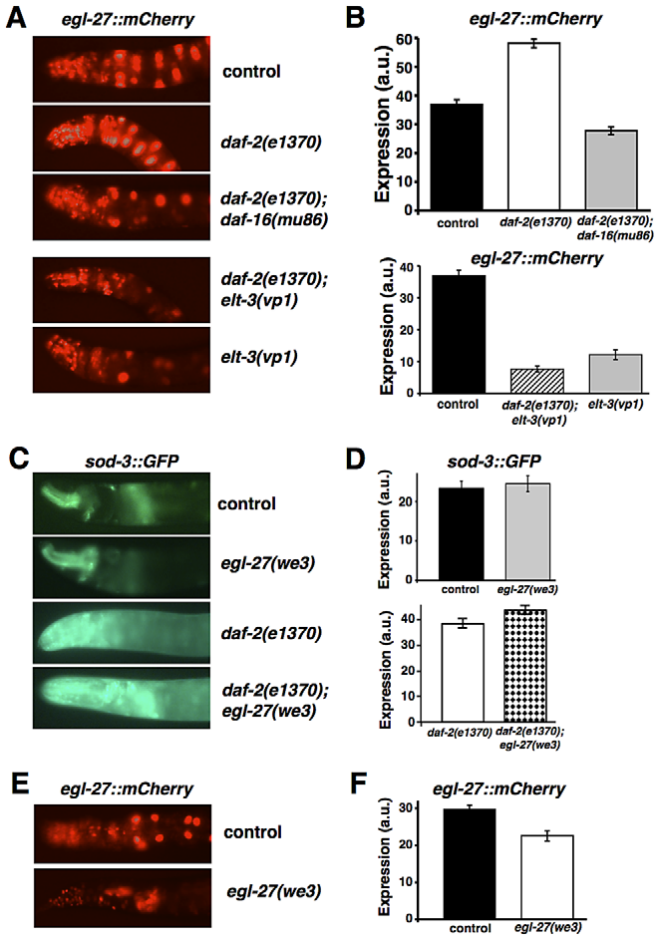
*egl-27* acts downstream of *daf-2*/ILR, *daf-16*/FOXO, and *elt-3*/GATA. (A) representative images of *egl-27::mCherry* hermaphrodites at day 2 of adulthood showing median expression levels for each group. (B) *egl-27* expression is increased in *daf-2(e1370)* mutants (p = 1.5×10^−11^ vs. control) and this increase is suppressed in *daf-2(e1370); daf-16(mu86)* double mutants (p = 2.9×10^−17^ vs. *daf-2(e1370)* worms). *egl-27* expression is reduced in *elt-3(vp1)* mutants (p = 2.3×10^−8^ vs. control) and the addition of *daf-2(e1370)* in *daf-2(e1370); elt-3(vp1)* double mutants is not sufficient to revert this reduction (p = 1.5×10^−18^ vs. control, p = 9.6×10^−29^ vs. *daf-2(1370)* worms). Quantification of intestinal expression for each group in arbitrary units was achieved by measuring fluorescence levels in 20 images using ImageJ. All values indicate mean expression and error bars represent SEM. All p-values are t-test p-values. (C,D) *egl-27* does not act upstream of *daf-16* or *elt-3*, as indicated by expression of a *sod-3::GFP* transcriptional reporter. (C) Representative images showing day 2 adult hermaphrodites grown at 20°C expressing *sod-3::GFP*. (D) Quantification of intestinal expression for each group in arbitrary units using imageJ to measure fluorescence from 15 images. *sod-3::GFP* expression is increased in *daf-2* mutants compared to wild-type. Levels of *sod-3::GFP* expression both in control and *daf-2(e1370)* worms are unaffected by *egl-27(we3)*. (E,F) *egl-27* regulates its own expression. (E) representative images showing *egl-27::mCherry* expression from day 2 adult hermaphrodites that were hatched at 20°C and grown at 15°C from day 1 of adulthood. (F) Quantification of intestinal expression for each group in arbitrary units using ImageJ to measure fluorescence intensity from 30 images. *egl-27* expression is increased in *egl-27(we3)* mutants (p = 0.0015).

The results presented above suggest that *egl-27* functions downstream of *daf-*2 in the IIS pathway. To test whether the IIS pathway can modulate *egl-27* expression, we examined whether *egl-27* expression is altered in *daf-2* mutants. Using fluorescence microscopy, we compared the intestinal expression of an integrated *egl-27::mCherry* transcriptional reporter in a strain with reduced *daf-2* activity to control worms in two day old adult, hermaphrodite worms (Figure 3A). We found that *egl-27* expression increased 58% in *daf-2(e1370)* mutants compared to control worms, providing molecular evidence that *egl-27* is regulated by *daf-2* in the IIS pathway (Figure 3B).

To further define where *egl-27* acts in the IIS pathway, we examined whether *egl-27* acts downstream or upstream of the IIS pathway modulator *daf-16*/FOXO. To do this, we tested whether a mutation in *daf-16* suppresses the increased levels of *egl-27* found in *daf-2* mutants (Figure 3A). We found that levels of *egl-27::mCherry* in *daf-2(e1370); daf-16(mu86)* double mutants are 2.1 fold lower than in *daf-2* mutants (Figure 3B). This indicates that *daf-16* is required for increased *egl-27* expression in *daf-2* mutants, which suggests that *egl-27*/GATA is regulated by *daf-16*/FOXO in the insulin signaling pathway. Further supporting this idea, we found a canonical DAF-16/FOXO binding element (GTAAACA) [28], [37] 614 bps upstream of the *egl-27* translational start site, suggesting that *egl-27* may be a direct target of DAF-16.

In addition to *daf-16*/FOXO, the GATA transcription factor *elt-3* modulates IIS, as *elt-3* expression is increased in *age-1* mutants with reduced IIS and *elt-3* is partially required for the longevity phenotype of *daf-2* mutants [29]. To determine whether *egl-27* acts downstream of the GATA transcription factor *elt-3*, we tested whether *egl-27* expression is affected by a null mutation in *elt-3* (Figure 3A). We found that levels of *egl-27::mCherry* are 3.0 fold lower in *elt-3(vp1)* mutants compared to control worms, suggesting that *egl-27* acts downstream of *elt-3* (Figure 3B). We next tested whether *elt-3* is required for increased levels of *egl-27* expression found in *daf-2* mutants. We found that levels of *egl-27::mCherry* in *daf-2(e1370); elt-3(vp1)* double mutants are 6.7 fold lower than in *daf-2* mutants and 4.8 fold lower than control worms (Figure 3B). Furthermore, levels of *egl-27::mCherry* in *daf-2(e1370); elt-3(vp1)* double mutants are similar to levels in *elt-3(vp1)* mutants (Figure 3B), suggesting that *elt-3* is necessary for heightened *egl-27* expression in the context of reduced IIS.

In the expression experiments above, we measured *egl-27::mCherry* expression in the anterior intestine. To determine whether *egl-27* is regulated in a tissue-specific manner, we also examined *egl-27* expression in the head region, which is composed of several different cell types – hypodermal, neuronal, muscle, and pharyngeal cells. We found that *egl-27::mCherry* levels are 35% higher in *daf-2(e1370)* mutants and 34% lower in *elt-3(vp1)* mutants compared to control worms. *egl-27::mCherry* levels are 37% lower in *daf-2(e1370); daf-16(mu86)* double mutants and 68% lower in *daf-2(e1370); elt-3(vp1)* double mutants compared to *daf-2(e1370)* worms (Figure S2A). These results suggest that IIS and *elt-3* regulate *egl-27* expression across multiple tissues, although the magnitude of this regulation may vary slightly across tissues.

We also determined whether *egl-27* regulation by IIS and *elt-3* GATA transcription occurs only during adulthood, or whether the same regulation occurs during development. To do this, we examined whether *egl-27* expression is affected by mutations in *daf-2* and *elt-3* at the L2 larval stage of development. We found that *egl-27* levels are 80% higher in *daf-2(e1370)* mutants and 3.1 fold lower in *elt-3(vp1)* mutants compared to control worms (Figure S2B). Because *egl-27* is regulated by *daf-2* and *elt-3* to approximately the same degree during development and adulthood, the genetic networks that regulate *egl-27* expression are likely developmental programs that persist into adulthood. To confirm our fluorescent microscopy results, we also examined how endogenous *egl-27* levels are affected in *daf-2* and *elt-3* mutants. Using qRT-PCR, we showed that *egl-27* levels are 67% higher in *daf-2(e1370)* mutants (p = 0.009) and 2.0 fold lower in *elt-3(vp1)* mutants (p = 0.003) compared to control worms during the L2 larval stage of development (Figure S2C, S2D).

To determine whether *egl-27* forms a feedback loop with *daf-16* and *elt-3*, we examined whether *egl-27* can act upstream of these regulators. To examine whether *egl-27* can regulate either DAF-16 or ELT-3 activity, we examined how reduction of *egl-27* affects levels of expression of *sod-3::GFP*, an established transcriptional reporter for DAF-16 [38], [39] and ELT-3 [29], [39] (Figure 3C). We found that levels of a *sod-3::GFP* transcriptional reporter are not significantly different between *egl-27(we3)* mutant worms and control worms (Figure 3D). However, DAF-16 activity is low in wild-type worms, so we examined whether reduction of *egl-27* affects levels of *sod-3::GFP* expression in *daf-2* mutants where DAF-16 is highly activated. Similar to previous reports [38], [39], we found using fluorescent microscopy that *sod-3* expression is 4.3 fold higher in *daf-2(e1370)* worms compared to control worms, and that increased *sod-3* expression is suppressed in *daf-2(e1370); daf-16(mu86)* double mutants (Figure S2E). We found that *sod-3* levels are equally high in *daf-2(e1370); egl-27(we3)* double mutants (Figure 3D). These results indicate that reduction of *egl-27* activity does not affect expression of *sod-3::GFP*, suggesting that neither DAF-16 nor ELT-3 activity are affected in *egl-27(we3)* mutants.

Finally, we examined whether *egl-27* can regulate its own expression. To do this, we examined how reduction of *egl-27* activity affects the expression of an *egl-27::mCherry* transcriptional reporter (Figure 3E). We found that *egl-27::mCherry* expression is reduced by 32% in *egl-27(we3)* mutants compared to control worms (Figure 3F). This suggests that *egl-27* activates its own expression in a feed-forward loop. Supporting this, we found several GATA-like binding motifs in the promoter region of *egl-27* (TTATC/GATAA 107 bps upstream, TATCA/TGATA 728 bps upstream, and CTTATCA/TGATAAG 800 bps upstream of the translational start site). These results suggest a role for GATA transcription factors such as ELT-3 or EGL-27 itself, in directly regulating *egl-27* expression.

### *egl-27* expression is induced by multiple stresses

**Figure 4:**
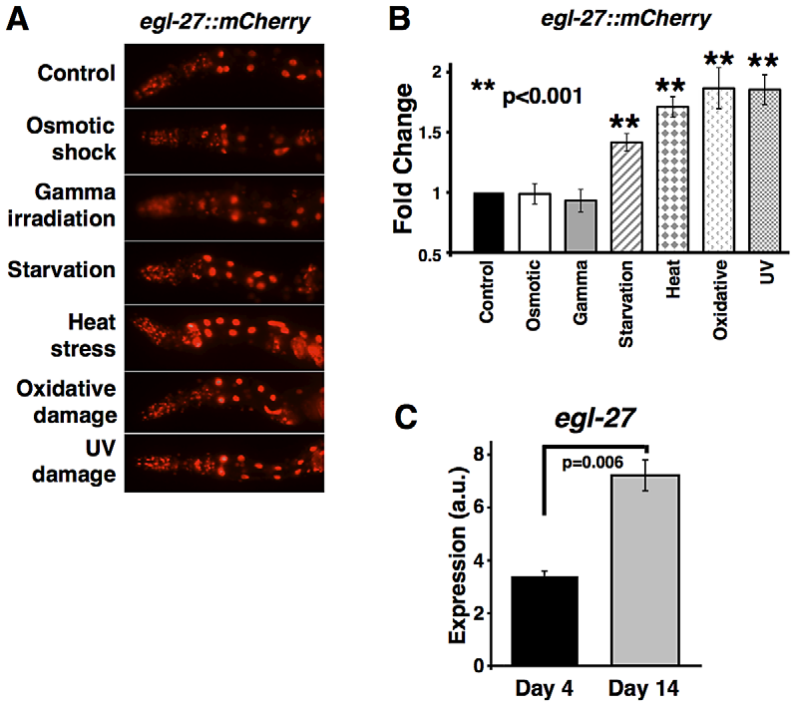
*egl-27* expression is induced in response to stress and aging. (A,B) *egl-27::mCherry* expression is induced in response to starvation, heat stress, oxidative stress, and UV stress. Day 1 adult hermaphrodites were exposed to indicated stresses and images of stressed and control worms were taken following stress exposure. (A) Representative images showing *egl-27::mCherry* expression in control and stressed worms. For all conditions, the worm with the median level of *egl-27::mCherry* expression is shown. (B) *egl-27::mCherry* expression was measured by quantification of fluorescence intensity of 15 images. Fold change in *egl-27::mCherry* expression for every condition was calculated in comparison to paired unstressed control. All values indicate mean expression and error bars represent SEM. (C) *egl-27* expression increases with age by qRT-PCR. *egl-27* expression levels were calculated using *act-1* as a normalization control.

In response to many types of stress, DAF-16/FOXO becomes activated [40], [41]. This leads to increased protection from the stress itself, and may also lead to increased levels of *egl-27* expression. According to this model, various types of stresses could also lead to changes in expression of *egl-27*, via activation of DAF-16/FOXO. To test this possibility, we exposed worms carrying an *egl-27::mCherry* transcriptional reporter to six different stresses (osmotic shock, gamma radiation, starvation, heat stress, oxidative damage, and UV damage). We then compared *egl-27::mCherry* expression under each stress condition to its expression in controls using fluorescent microscopy (Figure 4A). We found that *egl-27::mCherry* expression is induced after exposure to starvation, heat stress, oxidative damage, and UV stress (p value<0.001) (Figure 4B). Interestingly, none of the stresses increase *egl-27* expression by more than two-fold. This suggests that *egl-27* acts differently from canonical stress-induced genes, such as heat shock proteins, which are expressed at low levels under normal conditions but can be induced up to 100-fold following heat stress [42], [43].

As a control, we showed that the increase in *egl-27* expression is specific and not caused by a general increase in transcription in response to these stresses. We used fluorescence microscopy to measure expression levels of *myo-3::GFP*, a muscle-specific myosin gene (Figure S3A), and showed that its expression is not induced in response to starvation, heat stress, oxidative damage, or UV stress (Figure S3B).

### *egl-27* expression increases with age

To determine how *egl-27* expression changes during aging, we used qRT-PCR to measure *egl-27* expression in day 4 and day 14 adult worms. We found that *egl-27* expression increases two-fold between young and old worms (Figure 4C). This is consistent with previous work showing that stress genes such as heat shock proteins increase in expression as worms age before declining in expression in extremely old worms [18], [44]. These data suggest that *egl-27* expression, like the expression of other stress-related genes [18], [20], [21], [27], [45], increases in old worms.

### ChIP–seq analysis identifies EGL-27 binding sites

To better understand the mechanism by which *egl-27* promotes longevity and stress survival, we identified where EGL-27 binds in the genome. To do this, we prepared lysates of the *egl-27::GFP* worm strain described above, at the L2 larval stage of development. These lysates were used by the modENCODE consortium to generate binding site data for EGL-27, using a GFP antibody to immunoprecipate the GFP-tagged EGL-27. We chose to perform ChIP-seq using L2 larval stage worms rather than adult worms because the majority of datasets generated by modENCODE were for larval stages. Because we showed that *egl-27::GFP* can rescue the *egl-27(we3)* lethal mutant phenotype (Figure 1C), the sites that are bound by EGL-27::GFP are likely to be representative of the sites that are bound by endogenous EGL-27.

By examining ChIP-seq data from the modENCODE project, we identified 4113 DNA regions showing significant binding by EGL-27::GFP [46]. Previous work has shown that some DNA regions are bound by one or a few transcription factors (factor-specific) while other DNA sites are associated with a large number of transcription factors, and that the specific functions of each transcription factor are better defined by its factor-specific targets than by these redundantly bound targets [46]. Because we were interested in the factor-specific functions of EGL-27, we removed 2306 sites located within redundantly bound regions from further analysis. Of the factor-specific binding sites, 481 are located within gene promoters, defined as 1000 bps upstream to 500 bps downstream of a translational start site. 426 binding sites are located within exons and 466 binding sites are located within introns. 78 are located within 1000 bp downstream from the translational stop site. Finally, 516 are located in intergenic regions. Because we were interested in putative targets of EGL-27, we focused on the 481 peaks located within gene promoters.

**Figure 5:**
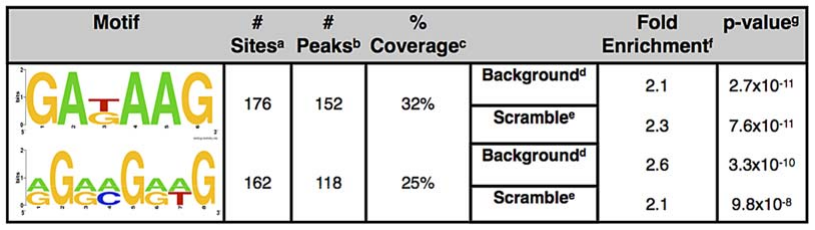
Motifs enriched in EGL-27 binding sites. ^a^ Total number of times motif is found in EGL-27 binding site. ^b^ Number of peaks that contain at least one instance of the motif. ^c^ % of peaks that contain at least one instance of the motif. ^d^ Promoter sequences randomly sampled from genome. ^e^ Scrambling of original peak sequence. ^f^ Number of peaks that contain at least one instance of motif/average number of peaks containing at least one instance of motif across 1000 iterations of background or scrambled peaks. ^g^ chi-square p-value comparing observed to average number of peaks containing at least one instance of motif across 1000 iterations of background or scrambled peaks.

To identify the consensus sites that may be directly bound by EGL-27, we examined the 481 ChIP-seq peaks that fall within gene promoters for the presence of enriched DNA motifs. We used the Gibbs sampling program BioProspector [47] to perform a *de novo* motif search on the center 100 bp sequence of EGL-27 ChIP-seq identified binding sites. The top 10 motifs found by the program are variations of two motifs: the GATA motif and a novel RGRMGRWG motif (Table S2). The GATA motif (GAKAAG) is found in 32% of EGL-27 target peaks and the novel RGRMGRWG motif is found in 25% of target peaks. Both are significant when compared to background sets consisting of randomly generated 100 bp sequences centered from 1000 bp upstream to 500 bp downstream of translational start sites (Figure 5). Both motifs are also significant when compared to scrambled sequence derived from EGL-27 peaks that preserve nucleotide frequencies (Figure 5).

The novel RGRMGRWG motif is not a consensus-binding site for any known class of transcription factors. However, this motif was previously identified as enriched in the promoters of differentially-expressed genes in insulin signaling *daf-2* mutants as well as sirtuin pathway mutants [48].

Since EGL-27 contains a GATA DNA-binding domain, the enrichment for GATA motifs in the EGL-27 binding sites supports its function as a GATA transcription factor. Previous studies have shown that GATA motifs are important for osmotic and pathogenic stress response [49]–[51] and aging [28], [29], which is consistent with our model that *egl-27* regulates stress and aging genes by binding the GATA motif.

### EGL-27 binds upstream of age- and stress-regulated genes

The 481 EGL-27 promoter peaks are located in the promoter regions of 501 unique genes (Table S3). We conducted gene ontology (GO) analysis [52], [53] on the set of 501 EGL-27 target genes in order to determine if the EGL-27 target genes are enriched for specific biological pathways. We found that EGL-27 targets are enriched for hypodermal genes that form the cuticle as well as intestinal genes involved in aromatic compound catabolic process (Table S4).

We examined whether target genes bound by EGL-27 significantly overlap genes that change expression with age. We obtained a list of 1132 age-regulated genes as previously defined by DNA microarrays [29]. Specifically, we computed the hypergeometric p-value for the overlap between the set of 501 EGL-27 target genes and the set of 1132 age-regulated genes. We found that 67 EGL-27 targets show age-dependent expression, which is a 2.8 fold enrichment over the number expected by chance (p = 9.5×10^−15^) (Table 1). Even though the ChIP-seq analysis was performed at the L2 larval stage of development, we were still able to find a strong enrichment for age-regulated genes. Interestingly, 61 of the 67 targets decline in expression with age (Figure S4) suggesting that increased levels of *egl-27* during normal aging are insufficient to prevent the age-dependent decline of these genes. Using GO analysis, we found that these 67 age-dependent EGL-27 targets are even further enriched for hypodermal genes that form the cuticle as well as several metabolic categories including aromatic amino acid family metabolic process, oxoacid metabolic process, and organic acid metabolic process (Table S4).

**Table 1:** Hypergeometic p-value for EGL-27 targets and 16 stress conditions and aging.

Condition	# EGL-27 targets	# Total genes	Fold Enrichment	p-value
***E. carotovora***	29	959	1.31	0.0837
***E. faecalis***	45	1161	1.68	4.54×10^−4^
***P. aeruginosa***	46	634	4.36	6.98×10^−17^
***P. luminescens***	52	1146	1.97	2.32×10^−6^
***S. aureus***	25	386	3.89	9.80×10^−9^
***S. marcesens***	31	1120	1.2	0.169
**Cry5b**	53	1012	3.15	2.08×10^−13^
**Cadmium**	55	992	3.33	6.72×10^−15^
**Ethanol**	13	219	2.84	7.36×10^−4^
**Silver**	64	1519	2.53	9.29×10^−12^
**Heat**	37	614	3.62	2.11×10^−11^
**Osmotic**	25	313	4.8	1.31×10^−10^
**Oxidative**	73	1243	3.53	5.61×10^−21^
**Gamma**	48	1946	1.48	4.29×10^−3^
**X-ray**	28	607	2.77	1.50×10^−6^
**Starvation**	127	3479	2.19	4.25×10^−18^
**Aging**	67	1132	2.83	9.51×10^−15^

Because we previously showed that *egl-27* expression is induced in response to several types of stress, we examined whether *egl-27* targets are differentially-expressed in response to different types of stress. To do this, we acquired published transcriptional responses to 16 different stress conditions involving response to six pathogens, six environmental stresses, and four toxins (Table S5). We then compared each set of genes that are differentially-expressed in that particular stress condition to the set of EGL-27 targets to determine whether the two sets significantly overlap by a hypergeometric test. Even though the ChIP-seq experiment for EGL-27 was not performed under stress conditions, we found that EGL-27 target genes are significantly (p<10^−5^) enriched for differentially-expressed genes from 11 of the 16 different stress conditions (Table 1). This supports the idea that *egl-27* is involved in the response to many types of stress.

### *egl-27* modulates heat stress gene expression in unstressed and heat-stressed worms

**Figure 6:**
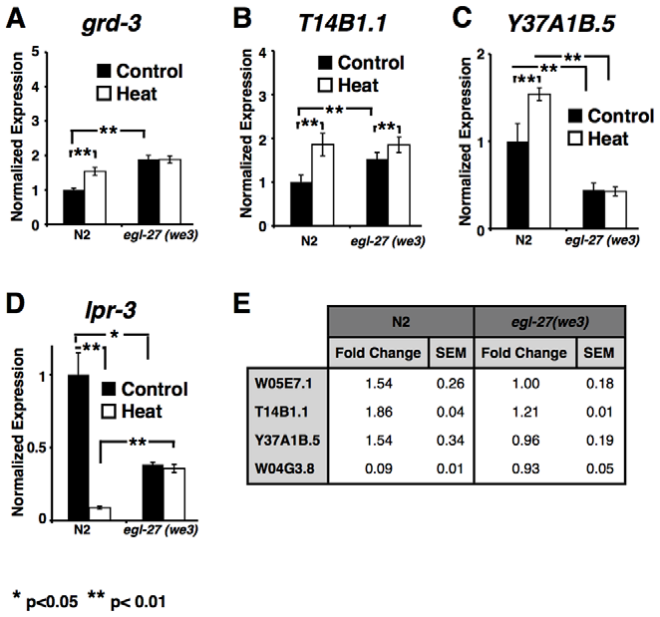
Effects of *egl-27(we3)* on expression of heat-stress gene expression. (A–D) qRT-PCR was used to measure levels of gene expression in wild-type (N2) and *egl-27(we3)* hermaphrodites at the L2 larval stage in unstressed (control) or heat-stressed (heat) conditions. *act-1* was used as a normalization control. * p<0.05, ** p<0.01 (A) *grd-3* (B) *T14B1.1* (C) *Y37A1B.5* (D) *lpr-3* (E) Fold change of expression between heat-stressed and unstressed worms was calculated for each of the four genes in both N2 and *egl-27(we3)*. SEM = standard error of the mean.

To better characterize how *egl-27* mediates heat-stress survival, we assessed how *egl-27* regulates heat-stress target genes. To do this, we examined the expression of four *egl-27* target genes (*grd-3*, *T14B1.1*, *Y37A1B.5*, and *lpr-3*) that were previously shown by DNA microarray experiments to be differentially expressed following heat stress [54]. We first wanted to see that expression of these genes change as expected after heat stress in wild-type worms. To do this, we extracted RNA from wild-type second larval stage worms that were exposed to 90 minutes of heat at 35°C followed by a 2-hour recovery. We then used qRT-PCR to determine how the expression of each gene changes in heat stressed worms compared to unstressed worms. Similar to the DNA microarray data, we found that *grd-3*, *T14B1.1*, and *Y37A1B.5* expression increases while *lpr-3* expression decreases after heat stress (Figure 6A–6D).

We examined how reduced *egl-27* activity affects target gene expression before heat stress. To do this, we compared expression levels of EGL-27 target genes in *egl-27(we3)* mutants to their expression levels in wild-type worms. We found that two genes (*grd-3* and *T14B1.1*) had higher expression while two genes (*Y37A1B.5* and *lpr-3*) had lower expression in *egl-27* loss-of-function worms than in wild-type worms (Figure 6A–6D). This shows that *egl-27* plays a role in modulating target gene expression under normal conditions when worms are unstressed.

Next, we assessed how reduction of *egl-27* activity affects the heat stress response of these four genes, where the heat stress response is defined as the ratio of expression after heat stress compared to before stress. We found that reduction of *egl-27* activity resulted in attenuation of the heat-induced changes of all four genes (Figure 6E). Interestingly, the reduced heat stress response is not caused by lower levels of induced expression, but rather by higher levels of basal expression. Expression of all four target genes was significantly altered in *egl-27* reduction-of-function mutants prior to heat stress (Figure 6A–6D). For example, in *egl-27* reduction-of-function worms, the expression of two targets (*grd-3* and *T14B1.1*) is induced even in pre-stressed animals. Although expression of these genes remains unchanged following stress, both pre-stress and post-stress expression levels in *egl-27* reduction-of-function worms are similar to their levels in post-stress wild-type worms. In the case of these two genes, *egl-27* may function to alter baseline expression levels in unstressed worms. However, expression levels of two other targets (*Y37A1B.5* and *lpr-3*) are significantly different in *egl-27* reduction-of-function worms compared to wild-type worms both pre- and post- heat stress. For these two genes, *egl-27* may function to alter baseline expression levels in unstressed worms and is required for additional activation of expression following heat stress. These results suggest that endogenous *egl-27* is required to reduce basal stress levels in wild-type worms.

To complement the reduction-of-function results, we examined how increased basal *egl-27* activity in the *egl-27::GFP* overexpression strain affects differential expression of *egl-27* in response to heat-stress (Figure S5). First, we used qRT-PCR to measure the combined RNA levels of *egl-27::GFP* and endogenous *egl-27*. As expected, the *egl-27::GFP* overexpression strain had both increased levels of combined *egl-27* expression before and after stress when compared to control worms (Figure S5E). Additionally, we observed that the heat stress response of *egl-27* is greater in *egl-27::GFP* worms compared to control worms (2.9 fold versus 2.2 fold respectively)(Figure S5E). This result suggests that worms with *egl-27::GFP* have increased levels of *egl-27* activity, which may make the expression of *egl-27* more sensitive to heat stress as part of a positive feedback loop.

However, the *egl-27::GFP* strain showed variable effects on the expression of EGL-27 target genes both before and after heat stress (Figure S5A–S5D). Expression of *grd-3* was unaffected by increased levels of *egl-27* before heat stress. Following heat stress, expression of *grd-3* was higher in the *egl-27::GFP* strain than in the control strain, such that induction levels were 1.8 fold in the *egl-27::GFP* strain compared to 1.2 fold in the control strain. Expression of *T14B1.1* was unaffected by increased levels of *egl-27* both before and after heat stress. Expression of *Y37A1B.5* was increased in *egl-27* gain-of-function worms before heat stress but repressed in these worms following heat stress. Finally, expression of *lpr-3* was 19-fold lower in *egl-27* gain-of-function worms before heat-stress compared to control worms and 3.6-fold lower following heat stress. Whereas heat stress causes this gene to decrease expression slightly in control worms, heat stress causes its expression to increase in the *egl-27::GFP* strain. While expression of *lpr-3* was significantly down-regulated following heat stress in wild-type worms, *lpr-3* levels were not significantly changed in transgenic control worms, underscoring the background differences that can exist between wild-type and transgenic animals. These data suggest that increased levels of *egl-27* affect the expression of some target genes both prior to and after heat stress.

## Discussion

We have shown that a GATA/MTA1 transcription factor [29]–[33], [55], *egl-27*, is an important mediator of stress response and longevity in *C. elegans*. Using binding site data from chromatin immunoprecipitation followed by ultra high throughput sequencing (ChIP-seq), we have shown that EGL-27 binds upstream of genes that are enriched for those that increase with age and that change in response to diverse stresses.

We examined whether EGL-27 has a beneficial or detrimental effect on longevity and stress response. We found that overexpression of *egl-27* increases both longevity as well as survival in response to heat stress. In contrast, *egl-27* mutants have a shortened lifespan and reduced survival in response to heat stress. Furthermore, reduction of *egl-27* activity suppresses the longevity phenotype as well as the heat and oxidative stress resistance phenotypes of *daf-2* mutants. These results suggest that *egl-27* may promote longevity through promoting stress resistance. This possibility is supported by other studies showing that increased expression of genes that confer resistance to specific stresses also extend lifespan. For example, increased levels of zebrafish *lysosyme* which confers antimicrobial defense [56], *heat shock factor 1* which confers heat resistance [5], [56], and *skn-1* which confers resistance to oxidative damage [6], [57], are all capable of extending lifespan when overexpressed in transgenic worms. Here, we identify a GATA transcription factor, *egl-27*, that plays a role in the response to multiple stresses and has a beneficial effect on longevity when overexpressed.

Altering levels of *egl-27* activity affects the expression of heat stress genes both in unstressed as well as heat-stressed worms. From these experiments, we infer that *egl-27* normally maintains a program to resolve cellular stress, and that altering levels of *egl-27* alters baseline stress levels in worms. Similar to this, a previous study showed that certain stress response genes are expressed at lower levels in stress-resistant *daf-2* mutants [58]. This suggests that that changes that alter baseline levels of stress can also alter baseline expression of stress response genes.

*egl-27* expression is regulated by a variety of stress pathways. We found that *egl-27* expression is induced by multiple stresses: heat stress, oxidative damage, UV irradiation, and starvation. Next, we showed that *egl-27* acts downstream of the GATA transcription factor *elt-3* and two IIS pathway components (IIS receptor *daf-2* and the FOXO transcription factor *daf-16*). *egl-27* expression is induced in long-lived *daf-2(e1370)* mutants and this induction is suppressed in *daf-2(e1370); daf-16(mu86)* and *daf-2(e1370); elt-3(vp1)* double mutants. These data support our finding that *egl-27(we3)* can fully suppress *daf-2(e1370)* longevity. Furthermore, previous work has shown that *elt-3* is a pro-longevity factor whose expression is confined mainly to hypodermal cells [29], [59], [60]. Our finding that *elt-3* can regulate *egl-27* expression in several tissue types including the intestine suggests that *elt-3* can affect gene expression in a cell non-autonomous manner.

Surprisingly, expression of *sod-3*, which acts as a readout for DAF-16 activity [38], [39], is unchanged in *daf-2(e1370); egl-27(we3)* double mutants compared to *daf-2(e1370)* single mutants, suggesting that activated DAF-16 is not sufficient for extended longevity in the absence of functional *egl-27*. Finally, we found that *egl-27* can regulate its own expression in a feed-forward loop. This evidence for auto-regulation supports the idea that *egl-27* may be involved in a complex circuit with feedback mechanism for regulating target gene expression.

Interestingly, *egl-27* expression increases with age in wild-type worms. Our finding that increased *egl-27* expression extends lifespan and improves stress resistance suggests that the way that *egl-27* expression changes during aging is beneficial for the organism. In contrast, previous studies have focused on age-dependent changes in expression that are detrimental for the organism. For example, the GATA transcription factor *elt-3* is an important regulator of the transcriptional changes that occur between young and old. *elt-3* declines in expression with age and low levels of *elt-3* have a deleterious effect on survival and stress response, suggesting that declining levels of *elt-3* may act as a driver of aging [29]. Another study found that NF-κB acts as a key regulator of age-dependent gene expression differences in nine types of human and mouse tissues [61]. NF-κB expression increases in old animals, and this increase is detrimental as blocking NK-κB in old skin results in a partial reversal of the aging transcriptome and more youthful skin [61], [62].

In contrast to *elt-3* in *C. elegans* and NF-κB in mice, our studies suggest that changes in *egl-27* expression during aging may act to improve stress response and to promote longevity. However, most EGL-27 binding targets from ChIP-seq experiments decline in expression with age, suggesting that this increased expression is insufficient to prevent the age-dependent decline of these genes. Because increased levels of *egl-27* extend lifespan, increased expression of *egl-27* in old worms appears to delay the aging process instead of causing it. Our work offers novel insight into the interplay between stress and aging, and suggests that aging is not simply a process of moving from an ideal young transcriptome to an inadequate old transcriptome. Rather, age-dependent changes in gene expression are likely comprised of a mix of beneficial, detrimental, and neutral changes.

## Materials and Methods

### Strains

All *C. elegans* strains (Table S6) were handled and maintained as described previously [63]. Genotyping primers are described in Table S7.

### Analysis of lifespan

Lifespan experiments were conducted as previously described [35], [64]. All experiments were done at 20°C unless otherwise noted. Age refers to days following adulthood and p-values were calculated using the log-rank (Mantel-Cox) method [65].

### Genotyping

*daf-2(e1370)* and *egl-27(we3)* are single base pair change mutants, so we used the tetra-primer ARMS-PCR procedure [66] to design 4 primers for each SNP of interest. *daf-16(mu86)* and *elt-3(vp1)* are deletions so we used a combination of 3 primers (two flanking the deleted region and one inside of the deleted region) to probe for the deletion. To generate DNA for genotyping, 1–10 worms were lysed in 1× PCR buffer with 1.5 mM MgCl_2_ and Protease K. A single PCR reaction was setup using this DNA and all (three or four) primers and then visualized on a 2% agarose gel. For *egl-27(we3)*, all reactions will produce a 396 bp product; mutant allele will produce a 213 bp product while wild-type allele will produce a 240 bp product. For *daf-2(e1370)*, all reactions will produce a 300 bp product; mutant allele will produce 155 bp product while wild-type allele will produce a 203 bp product. For *daf-16(mu86)*, mutant allele will produce a 400 bp product while wild-type allele will produce a 634 bp product. For *elt-3(vp1)*, mutant allele will produce a 145 bp product while wild-type allele will produce a 401 bp product. Primers and temperatures are detailed in Table S7.

### Rescue experiment

Worms carrying the integrated transgene *egl-27::GFP* (OP177) were crossed to *egl-27(we3)* (JA1194) to generate *egl-27::GFP; egl-27(we3)* (SD1751) in the F2 generation, which was identified by PCR genotyping (genotyping primers for *egl-27(we3)* detailed in Table S7; methods for single nucleotide genotyping above). N2, *egl-27(we3)*, and *egl-27::GFP; egl-27(we3)* worms were synchronized using hypochlorite and grown to day 1 of adulthood. 10 hermaphrodites from each strain were individually placed onto NGM plates seeded with *E. coli* and allowed to lay eggs for 1 hour. The adult worms were removed and the number of eggs counted. The numbers of hatched worms were scored 1 day, 2 days (not shown), and 6 days (Figure 1C) after the beginning of egg laying. % survival was computed as the percentage of hatched worms divided by the number of total eggs. Error bars represent the standard error between the 10 replicates.

### Imaging and quantification of Cherry and GFP expression

To quantify mCherry and GFP expression, we imaged at least 15 worms for each condition at 20× using a Zeiss Axioplan microscope. Images were analyzed using ImageJ [67].

### Stress assays

All assays were performed using day 1 adult hermaphrodites. In all cases, *E. coli* refers to the OP50 strain. Control worms were always transferred the same number of times, and imaged at the same time as experimental worms. All strains used for imaging and survival assays are detailed in Table S6.

#### Osmotic shock

Worms were transferred to high salt NGM plates (200 mM NaCl) seeded with concentrated *E. coli* for 90 minutes and imaged immediately.

#### Gamma radiation

Worms were transferred to NGM plates without *E. coli* and irradiated with a ^137^Cs source at 40 Gy as described previously [29]. Worms were then transferred to NGM plates seeded with *E. coli* and allowed to recover for 24 hours before imaging. Control worms were transferred to unseeded NGM plates, allowed to recover on seeded NGM plates, and imaged at the same time as experimental worms.

#### Starvation

Worms were transferred to unseeded NGM plates for 2 hours and imaged immediately.

#### UV radiation

Worms were transferred to NGM plates without *E. coli* and irradiated with 30 J/m^2^ UV using a UV Stratalinker (Stratagene) as described previously [68]. Worms were then transferred to NGM plates seeded with *E. coli* and allowed to recover for 5 hours before imaging. Control worms were transferred to unseeded NGM plates, allowed to recover on seeded NGM plates, and imaged at the same time as experimental worms.

#### Heat stress

Worms were incubated at 35°C for 90 minutes, and allowed to recover at 20°C for 1 hour before imaging. For the survival assay, worms were incubated at 35°C for 8 hours before scoring.

#### Oxidative stress

Worms were transferred to NGM plates containing 10 mM Paraquat, and imaged 24 hours later. For survival assay, worms were transferred onto NGM plates containing 10 mM Paraquat supplemented with 30 mM FuDR to prevent growth of progeny. Plates were seeded with concentrated *E. coli*. Death was scored as described above.

### Identification of ChIP–seq targets

Two independent cultures of worms expressing *egl-27::GFP* (OP177) were synchronized using sodium hypochlorite to isolate eggs followed by hatching in S basal overnight. Arrested L1 stage larva were collected and grown on NGM plates seeded with *E. coli* until mid L2 stage. ChIP-seq was performed by the modENCODE consortium [46], [69], [70]. The program PeakSeq [71] was used to identify EGL-27::GFP binding sites (q value<0.00001). Peaks bound by five or more out of the original 23 transcription factors were removed from further analysis. Significant, factor-specific peaks were then compared to the *C. elegans* genome to identify putative EGL-27 target genes. A gene is identified as a target if the center of a peak occurs 1.5 kb upstream or 500 bp downstream of its translational start site.

### Motif analysis

The center 100 bp sequences from the 481 promoter peaks were filtered for low complexity regions using RepeatMasker [72] and then submitted to BioProspector to identify overrepresented *cis* regulatory sequences [47]. To eliminate motifs or amino acid distributions that are generally enriched in all promoters, a random set containing 481 masked 100 bp promoter sequences was submitted to BioProspector as background input. We report the highest 10 motifs from BioProspector (Table S2), but since only 2 are unique, we ran further analysis on these two.

To determine the fold enrichment and probability of these two motifs occurring in our dataset compared to the probability expected by chance, 1000 datasets containing 481 randomly-generated 100 bp promoter sequences (to match the number of promoter peaks) were created. We scored the number of times these two motifs were found in our promoter sequences and the average number of times they were found in each of the 1000 background datasets. An enrichment factor for each motif was calculated using (# of ChIP-seq peaks containing at least one instance of the motif)/(average # of background peaks containing at least one instance of the motif). A chi-squared p-value for each motif was calculated using the # of ChIP-seq peaks containing at least one instance of the motif as observed and # of background peaks containing at least one instance of the motif as expected (Figure 5).

As a second control, we generated a set containing the 481 original sequences but scrambled the order of the nucleotides in order to create a random sequence while preserving the nucleotide frequency. We generated 1000 iterations of this set of 481 scrambled sequences and for each motif, scored the average number of times they were found in this scrambled sequences. Again, we calculated an enrichment factor and a p-value for each motif in comparison to the scrambled sequences (Figure 5).

### Gene Ontology analysis

EGL-27 target genes and age-regulated EGL-27 target genes were submitted to GOrilla [52], [53] for Gene Ontology (GO) analysis. A background gene list was also submitted. For EGL-27 target genes, the background list consisted of all annotated *C. elegans* genes. For age-regulated EGL-27 target genes, the background list consisted of all genes represented on the Stanford *C. elegans* microarray platform. GOrilla outputs a FDR q-value and an enrichment score for each GO category. The FDR q-value is the hypergeometric p-value corrected for multiple hypotheses testing using the Benjamini and Hochberg method [73]. Enrichment score is computed as (# of inputted genes in GO category/# of total inputted genes)/(# of genes in each GO category/# of background genes) [52], [53].

### Comparison with aging and stress microarrays

Age-regulated genes were obtained from [29] and probe IDs were remapped to WormBase gene IDs (WS228) using annotations from Stanford Microarray Database. Probes that could not be mapped to gene IDs were removed from further analysis. Differentially-expressed stress response genes for each of 16 stress conditions were obtained from publications detailed in Table S5. When probe names were supplied, probe IDs were remapped to WormBase gene IDs (WS228) using annotations from Affymetrix, Stanford Microarray Database, and Washington University Genome Sequencing Center. Probes that match multiple genes were assigned all associated gene IDs. Probes that could not be mapped to gene IDs were removed from further analysis. If only gene names were supplied, gene IDs were filtered using WormBase gene annotations (WS228). Unmatched probe IDs or gene IDs were removed from further analysis.

To determine whether EGL-27 target genes were significantly enriched for age-regulated or stress-response gene sets, we first determined the number of EGL-27 targets that are significantly differentially-expressed in each age or stress response set. For each comparison, the background gene count is the number of genes included in the platform for that microarray. Using this number, we computed, both a hypergeometric p-value and an enrichment score for each comparison, where enrichment score is defined as (# of EGL-27 targets/# of differentially expressed genes)/(# EGL-27 targets/# of background genes).

### Quantitative RT–PCR

N2, *egl-27(we3)*, control, and *egl-27::GFP (OP177)* worms (full genotypes and strain information in Table S6) were synchronized using hypochlorite to isolate eggs followed by hatching in S basal overnight. Arrested L1 stage larva were collected and grown on NGM plates seeded with *E. coli* until mid-L2 stage before splitting into an experimental and control set. Experimental worms were then incubated at 35°C for 90 minutes and allowed to recover at 20°C for 2 hours before collecting in Trizol (Invitrogen). Control worms were kept at 20°C and collected in Trizol at the same time. Total RNA was isolated using Trizol reagent, treated with DNaseI to degrade genomic DNA, and purified using RNeasy kit (Qiagen). cDNA was synthesized using oligo dT primers and SuperScript II First Strand Kit (Invitrogen). qPCR reactions were performed using RT^2^SYBR Green qPCR Mastermix (Qiagen) and the 7900HT Fast Real-Time PCR System (ABI). Melting curve analysis was performed with each primer pair to ensure that quantification is the result of only one product. A serial dilution was performed for each primer pair to generate a standard curve. *act-1* was used as an internal control to normalize expression levels as previously described [74], [75]. All primers are detailed in Table S7.

## Supporting Information

Figure S1 The cold-sensitive effect of *egl-27(we3)* allele has only a mild effect on longevity. (A) *egl-27(we3)* suppresses *daf-2(e1370)* longevity at all temperatures. All worms were hatched at 20°C and shifted to the indicated temperatures at day 2 of adulthood. *daf-2(e1370)* (15°C): n = 153, mean lifespan = 34.2, median lifespan = 35, 95% mortality lifespan = 44.4. *daf-2(e1370); egl-27(we3)* (15°C): n = 168, mean lifespan = 17.6, median lifespan = 12, 95% mortality = 43. *daf-2(e1370)* (20°C): n = 162, mean lifespan = 31.3, median lifespan = 31, 95% mortality = 47. *daf-2(e1370); egl-27(we3)* (20°C): n = 134, mean lifespan = 19.8, median lifespan = 16, 95% mortality = 43.4. *daf-2(e1370)* (25°C): n = 216, mean lifespan = 21.8, median lifespan = 22, 95% mortality = 34. *daf-2(e1370); egl-27(we3)* (25°C): n = 113, mean lifespan = 14.0, median lifespan = 13, 95% mortality = 23.2. (B,C) Three strains overexpressing the *we3* allele of *egl-27* extend lifespan compared to control. (B) Worms were hatched at 15°C and shifted to 20°C at day 2 of adulthood. Control: n = 61, mean lifespan = 14.3, median lifespan = 13, 95% mortality = 23. *egl-27(we3) OE #1*: n = 128, mean lifespan = 16.9, median lifespan = 16, 95% mortality = 30. *egl-27(we3) OE #2*: n = 106, mean lifespan = 17.5, median lifespan = 16.5, 95% mortality = 30.8. *egl-27(we3) OE #3*: n = 137, mean lifespan = 18.3, median lifespan = 17, 95% mortality = 30. (C) Worms were hatched at 15°C and maintained at 15°C throughout adulthood. Control: n = 54, mean lifespan = 21.4, median lifespan = 19, 95% mortality = 38. *egl-27(we3) OE #1*: n = 131, mean lifespan = 23.1, median lifespan = 21, 95% mortality = 38. *egl-27(we3) OE #2*: n = 92, mean lifespan = 23.9, median lifespan = 23, 95% mortality = 38. *egl-27(we3) OE #3*: n = 99, mean lifespan = 23.4, median lifespan = 21, 95% mortality = 37.1. (D) qRT-PCR analysis of *egl-27* levels in *egl-27* overexpression lines. *act-1* was used as a normalization control.
(TIF)

Figure S2 *egl-27* acts downstream of IIS and *elt-3* GATA transcription in multiple tissues and stages. (A) ImageJ quantification of *egl-27::mCherry* expression in the head region of 20 day 2 hermaphrodites shows that *egl-27* expression is increased in *daf-2(e1370)* mutants (p = 6.6×10^−6^ compared to control worms) and that this increase is suppressed in *daf-2(e1370); daf-16(mu86)* double mutants (p = 5.5×10^−7^ compared to *daf-2(e1370)* mutants). *egl-27* expression is reduced in both *elt-3(vp1)* mutants (p = 4.9×10^−6^ compared to control) and *daf-2(e1370); elt-3(vp1)* double mutants (p = 0.0016 compared to control, p = 8.6×10^−12^ compared to *daf-2(e1370)* mutants). (B) ImageJ quantification of *egl-27::mCherry* expression in the anterior intestinal region of 20 L2 larval stage hermaphrodites shows that *egl-27* expression is increased in *daf-2(e1370)* mutants (p = 1.6×10^−5^ compared to control) and reduced in *elt-3(vp1)* mutants (p = 1.8×10^−5^ compared to control). (C, D) qRT-PCR shows that endogenous *egl-27* levels are altered in *daf-2* and *elt-3* mutants in hermaphrodites at the L2 stage of development. *act-1* was used as a normalization control. (C) *egl-27* expression is increased in *daf-2(e1370)* mutants compared to wild-type worms (p = 0.0088) (D) *egl-27* expression is reduced in *elt-3(vp1)* mutants compared to wild-type worms(p = 0.0029) (E) *sod-3::GFP* is highly activated in *daf-2* mutants. Quantification is of intestinal expression for each group in arbitrary units using ImageJ to measure fluorescence from 15 images. Levels of *sod-3::GFP* transcriptional reporter are 4.3 fold higher in *daf-2(e1370)* worms compared to control worms, and this increase is abolished in *daf-2(e1370); daf-16(mu86)* double mutants.
(TIF)

Figure S3 *myo-3* expression levels are not affected by most stresses. (A,B) *myo-3::GFP* serves as a negative control to show that gene expression is not generally induced under conditions of starvation, heat stress, oxidative stress, and UV damage. *myo-3::GFP* expression is slightly induced after osmotic stress. (A) representative images showing *myo-3::GFP* expression in control and stressed worms. For all conditions, the worm with median levels of *myo-3* expression is shown. (B) *myo-3::GFP* expression was measured by quantification of fluorescence intensity of 15 images. Fold change in *myo-3::GFP* expression for every condition was calculated in comparison to paired unstressed control.
(TIF)

Figure S4 Age-dependent EGL-27 targets primarily decline in expression with age. Heatmap showing expression changes of 67 age-dependent EGL-27 target genes during aging. Expression change for a given day X is represented as −log_2_(Expression on day X/Expression on day 4). Expression data from [29].
(TIF)

Figure S5 *egl-27* regulation of heat stress gene expression. (A–E) qRT-PCR measured gene expression in control and *egl-27::GFP (OP177)* overexpression worms in unstressed (control) or heat stressed (heat) conditions. *act-1* was used as a normalization control. (A) *grd-3* (B) *T14B1.1* (C) *Y37A1B.5* (D) *lpr-3* (E) *egl-27*.
(TIF)

Table S1 Additional lifespan data.
(DOCX)

Table S2 Top ten motifs in EGL-27 binding sites found by BioProspector.
(DOCX)

Table S3 List of EGL-27 targets.
(XLSX)

Table S4 Top GO categories enriched in EGL-27 targets.
(DOCX)

Table S5 Description of all stress microarray datasets used.
(DOCX)

Table S6 Strains.
(DOCX)

Table S7 Primers.
(DOCX)
