# Supplementary figures and images for: The GATA Transcription Factor egl-27 Delays Aging by Promoting Stress Resistance in Caenorhabditis elegans
Source: PLoS Genet. 2012 Dec 13;8(12):e1003108. doi: 10.1371/journal.pgen.1003108 (PMC3521710; doi:10.1371/journal.pgen.1003108)

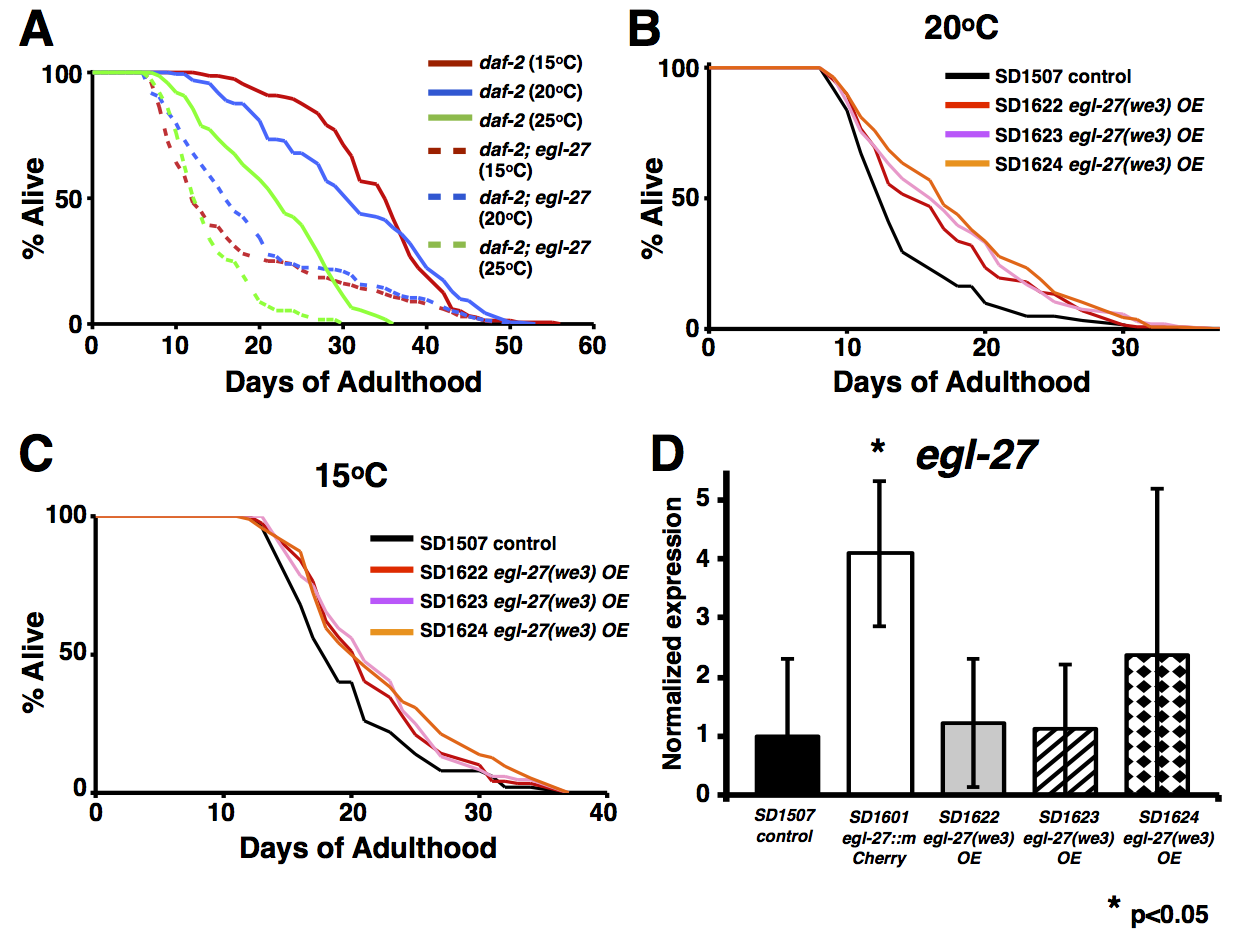

Supplement: Figure S1 — The cold-sensitive effect of egl-27(we3) allele has only a mild effect on longevity. (A) egl-27(we3) suppresses daf-2(e1370) longevity at all temperatures. All worms were hatched at 20°C and shifted to the indicated temperatures at day 2 of adulthood. daf-2(e1370) (15°C): n = 153, mean lifespan = 34.2, median lifespan = 35, 95% mortality lifespan = 44.4. daf-2(e1370); egl-27(we3) (15°C): n = 168, mean lifespan = 17.6, median lifespan = 12, 95% mortality = 43. daf-2(e1370) (20°C): n = 162, mean lifespan = 31.3, median lifespan = 31, 95% mortality = 47. daf-2(e1370); egl-27(we3) (20°C): n = 134, mean lifespan = 19.8, median lifespan = 16, 95% mortality = 43.4. daf-2(e1370) (25°C): n = 216, mean lifespan = 21.8, median lifespan = 22, 95% mortality = 34. daf-2(e1370); egl-27(we3) (25°C): n = 113, mean lifespan = 14.0, median lifespan = 13, 95% mortality = 23.2. (B,C) Three strains overexpressing the we3 allele of egl-27 extend lifespan compared to control. (B) Worms were hatched at 15°C and shifted to 20°C at day 2 of adulthood. Control: n = 61, mean lifespan = 14.3, median lifespan = 13, 95% mortality = 23. egl-27(we3) OE #1: n = 128, mean lifespan = 16.9, median lifespan = 16, 95% mortality = 30. egl-27(we3) OE #2: n = 106, mean lifespan = 17.5, median lifespan = 16.5, 95% mortality = 30.8. egl-27(we3) OE #3: n = 137, mean lifespan = 18.3, median lifespan = 17, 95% mortality = 30. (C) Worms were hatched at 15°C and maintained at 15°C throughout adulthood. Control: n = 54, mean lifespan = 21.4, median lifespan = 19, 95% mortality = 38. egl-27(we3) OE #1: n = 131, mean lifespan = 23.1, median lifespan = 21, 95% mortality = 38. egl-27(we3) OE #2: n = 92, mean lifespan = 23.9, median lifespan = 23, 95% mortality = 38. egl-27(we3) OE #3: n = 99, mean lifespan = 23.4, median lifespan = 21, 95% mortality = 37.1. (D) qRT-PCR analysis of egl-27 levels in egl-27 overexpression lines. act-1 was used as a normalization control. (TIF) [file pgen.1003108.s001.tif]

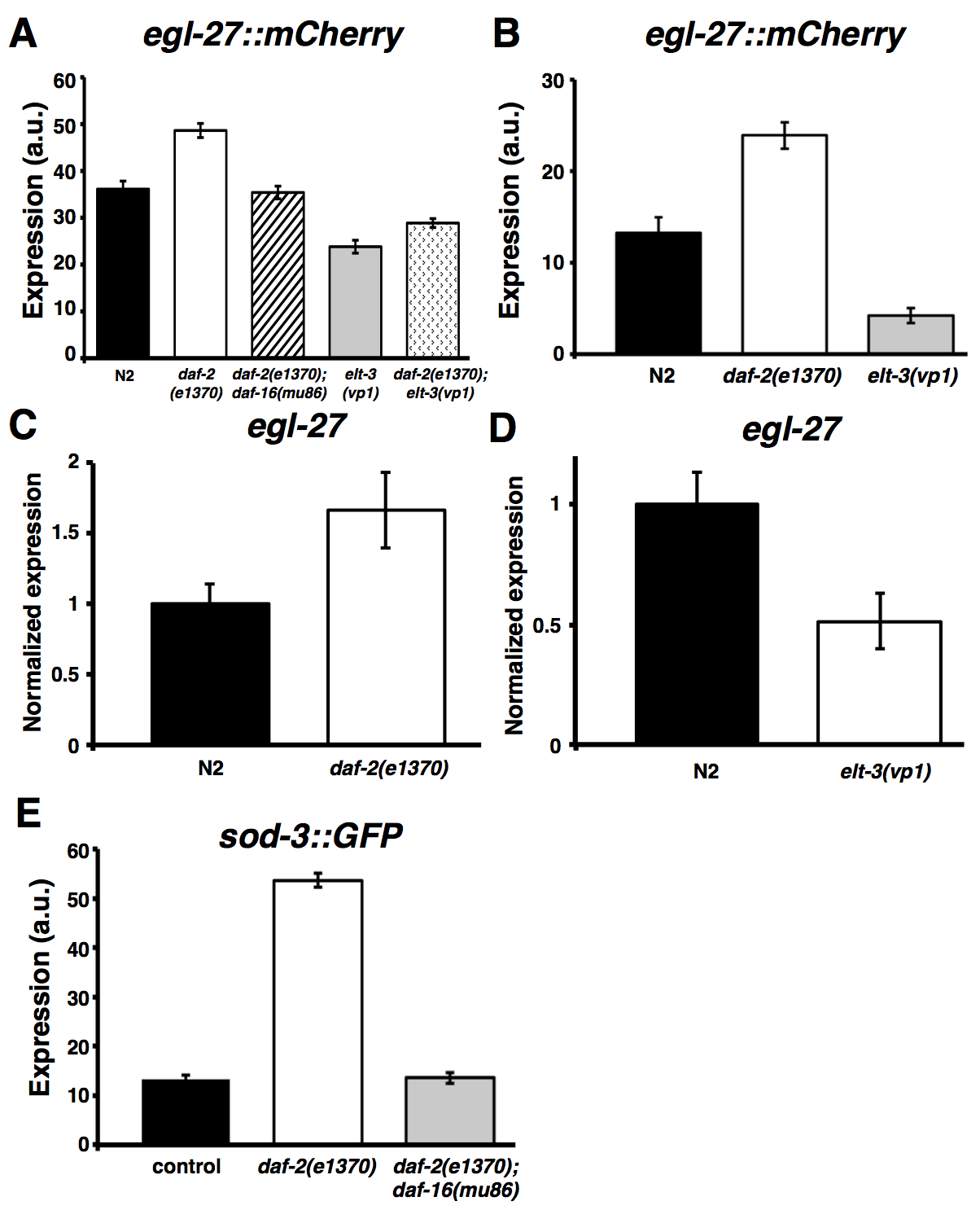

Supplement: Figure S2 — egl-27 acts downstream of IIS and elt-3 GATA transcription in multiple tissues and stages. (A) ImageJ quantification of egl-27::mCherry expression in the head region of 20 day 2 hermaphrodites shows that egl-27 expression is increased in daf-2(e1370) mutants (p = 6.6×10^−6^ compared to control worms) and that this increase is suppressed in daf-2(e1370); daf-16(mu86) double mutants (p = 5.5×10^−7^ compared to daf-2(e1370) mutants). egl-27 expression is reduced in both elt-3(vp1) mutants (p = 4.9×10^−6^ compared to control) and daf-2(e1370); elt-3(vp1) double mutants (p = 0.0016 compared to control, p = 8.6×10^−12^ compared to daf-2(e1370) mutants). (B) ImageJ quantification of egl-27::mCherry expression in the anterior intestinal region of 20 L2 larval stage hermaphrodites shows that egl-27 expression is increased in daf-2(e1370) mutants (p = 1.6×10^−5^ compared to control) and reduced in elt-3(vp1) mutants (p = 1.8×10^−5^ compared to control). (C, D) qRT-PCR shows that endogenous egl-27 levels are altered in daf-2 and elt-3 mutants in hermaphrodites at the L2 stage of development. act-1 was used as a normalization control. (C) egl-27 expression is increased in daf-2(e1370) mutants compared to wild-type worms (p = 0.0088) (D) egl-27 expression is reduced in elt-3(vp1) mutants compared to wild-type worms(p = 0.0029) (E) sod-3::GFP is highly activated in daf-2 mutants. Quantification is of intestinal expression for each group in arbitrary units using ImageJ to measure fluorescence from 15 images. Levels of sod-3::GFP transcriptional reporter are 4.3 fold higher in daf-2(e1370) worms compared to control worms, and this increase is abolished in daf-2(e1370); daf-16(mu86) double mutants. (TIF) [file pgen.1003108.s002.tif]

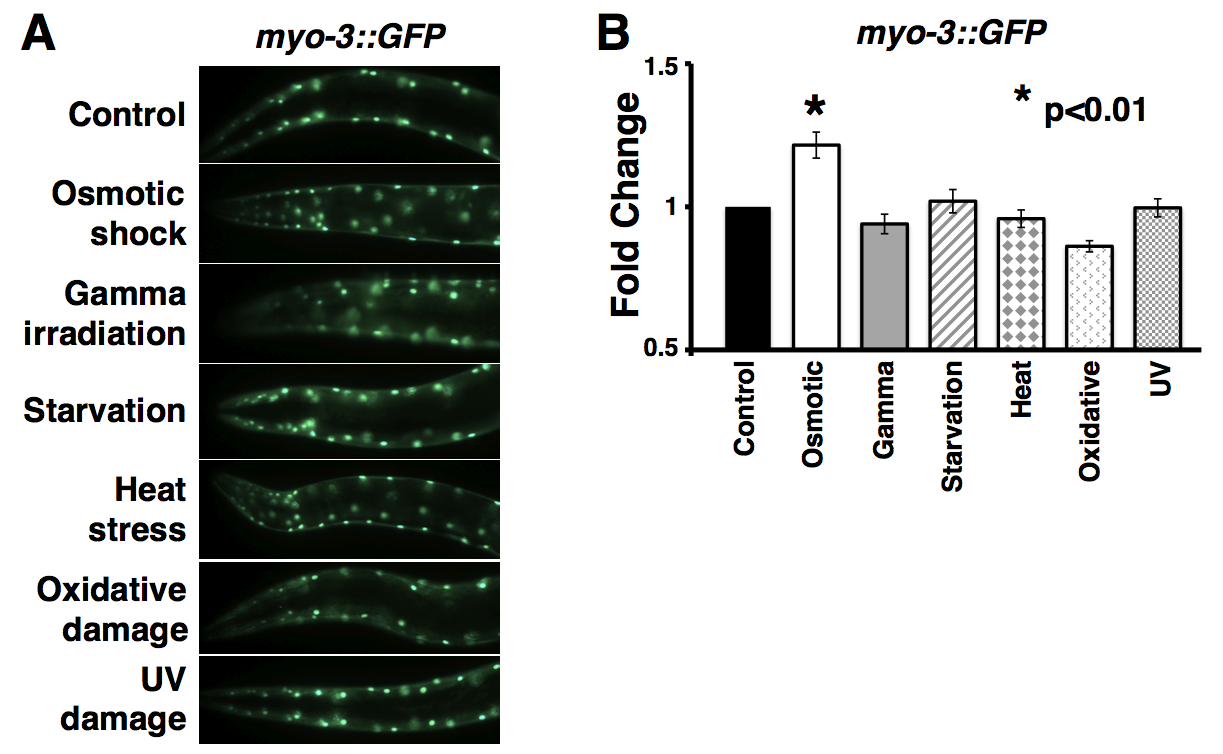

Supplement: Figure S3 — myo-3 expression levels are not affected by most stresses. (A,B) myo-3::GFP serves as a negative control to show that gene expression is not generally induced under conditions of starvation, heat stress, oxidative stress, and UV damage. myo-3::GFP expression is slightly induced after osmotic stress. (A) representative images showing myo-3::GFP expression in control and stressed worms. For all conditions, the worm with median levels of myo-3 expression is shown. (B) myo-3::GFP expression was measured by quantification of fluorescence intensity of 15 images. Fold change in myo-3::GFP expression for every condition was calculated in comparison to paired unstressed control. (TIF) [file pgen.1003108.s003.tif]

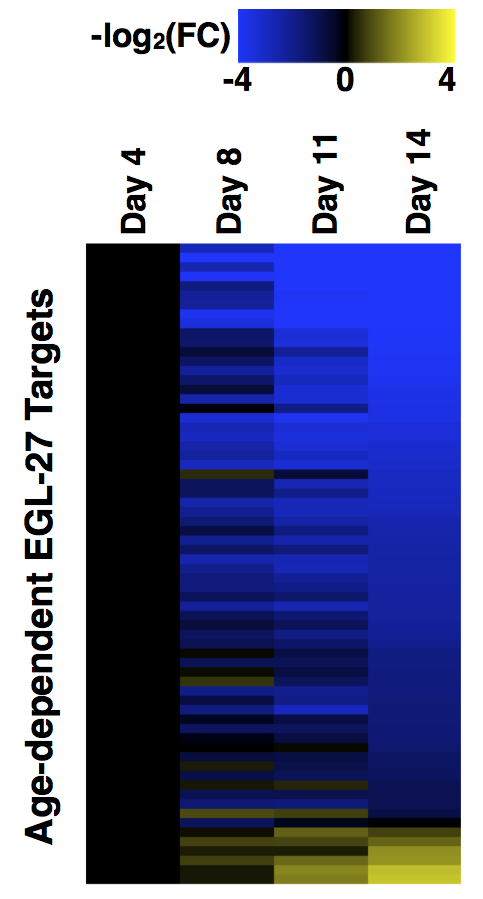

Supplement: Figure S4 — Age-dependent EGL-27 targets primarily decline in expression with age. Heatmap showing expression changes of 67 age-dependent EGL-27 target genes during aging. Expression change for a given day X is represented as −log2(Expression on day X/Expression on day 4). Expression data from [29]. (TIF) [file pgen.1003108.s004.tif]

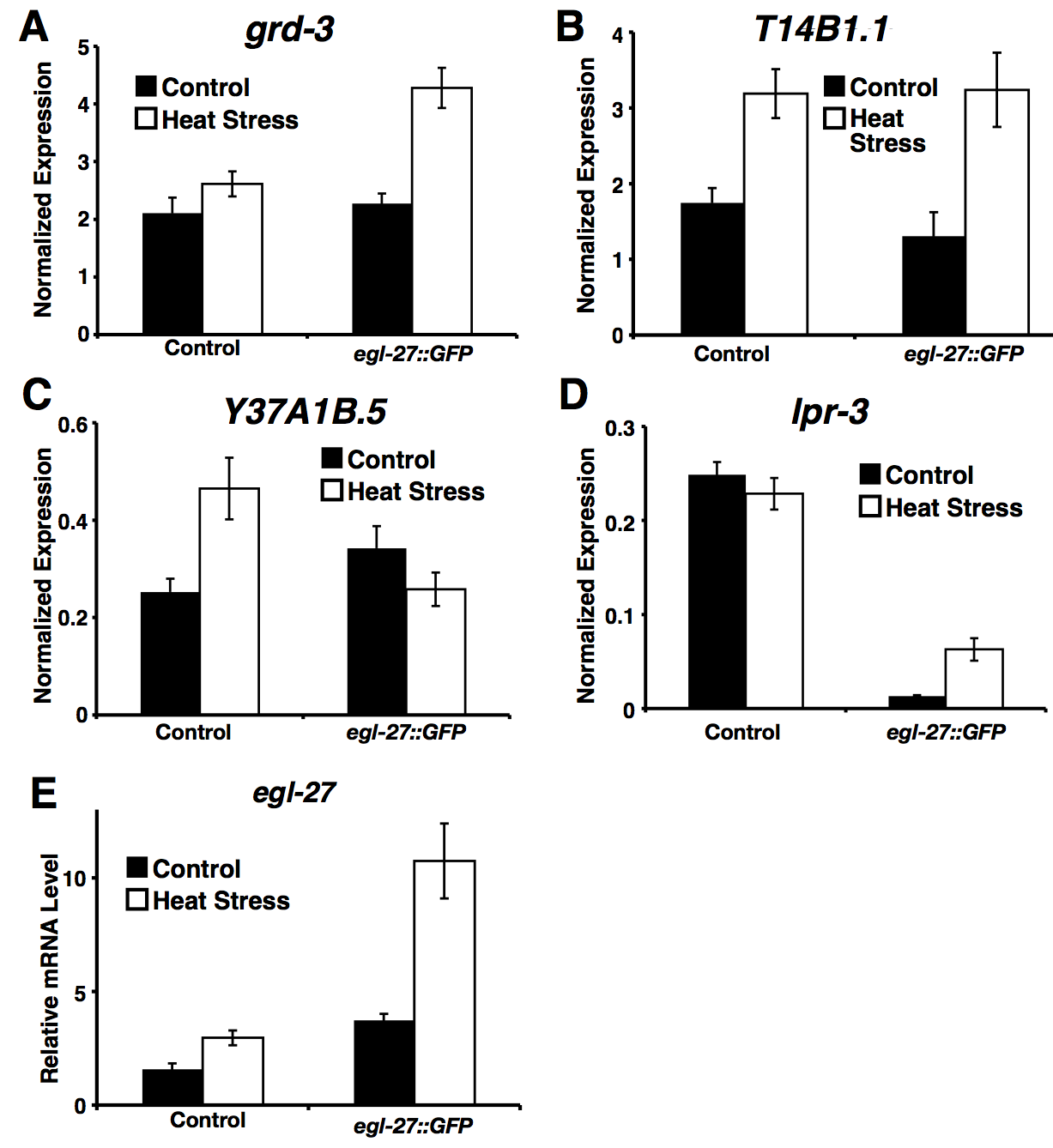

Supplement: Figure S5 — egl-27 regulation of heat stress gene expression. (A–E) qRT-PCR measured gene expression in control and egl-27::GFP (OP177) overexpression worms in unstressed (control) or heat stressed (heat) conditions. act-1 was used as a normalization control. (A) grd-3 (B) T14B1.1 (C) Y37A1B.5 (D) lpr-3 (E) egl-27. (TIF) [file pgen.1003108.s005.tif]

**Table S2. Top ten motifs in EGL-27 binding sites found by BioProspector.**

| **Motif** | **Score** | **# Sites** |
| --- | --- | --- |
| 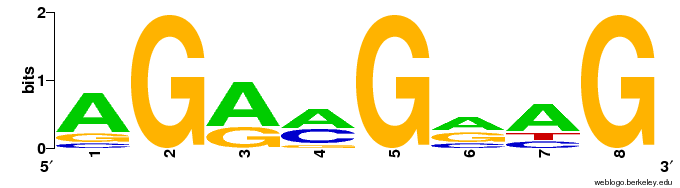 | 5.031 | 274 |
| 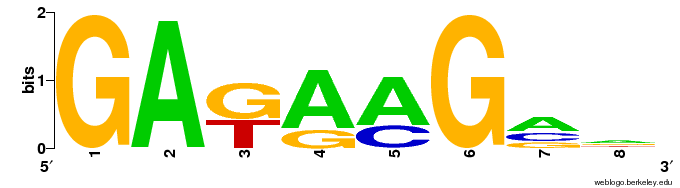 | 5.024 | 272 |
| 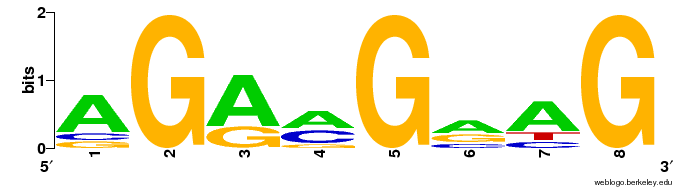 | 5.020 | 276 |
| 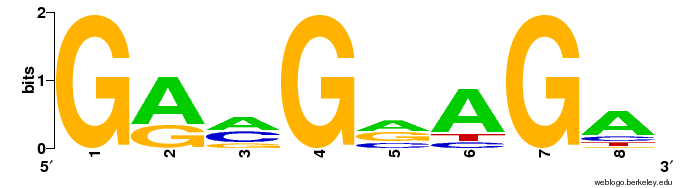 | 4.905 | 277 |
| 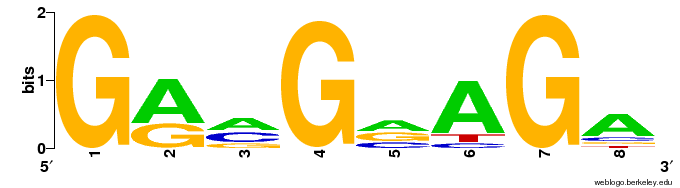 | 4.896 | 275 |
| 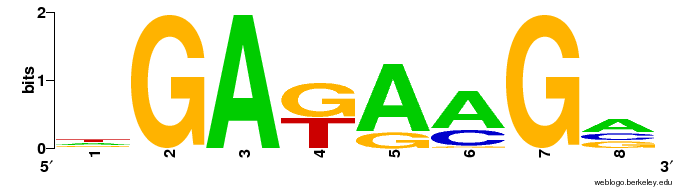 | 4.871 | 287 |
| 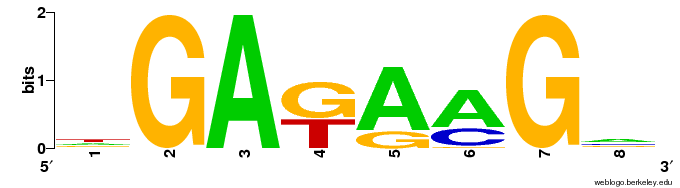 | 4.871 | 295 |
| 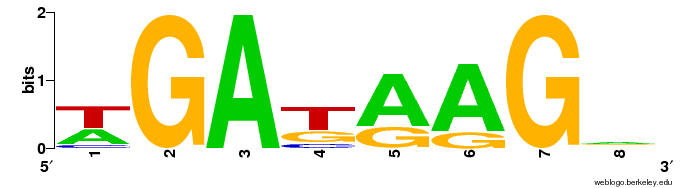 | 4.869 | 264 |
| 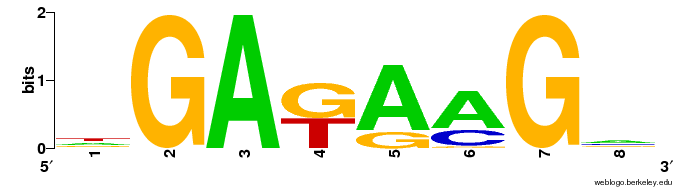 | 4.838 | 301 |
| 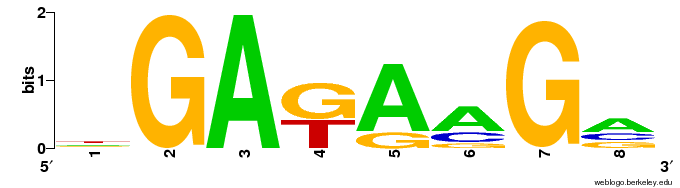 | 4.837 | 300 |

Supplement: Table S2 — Top ten motifs in EGL-27 binding sites found by BioProspector. (DOCX) [file pgen.1003108.s007.docx]
