# Supplementary material for: The GATA Transcription Factor egl-27 Delays Aging by Promoting Stress Resistance in Caenorhabditis elegans
Source: PLoS Genet. 2012 Dec 13;8(12):e1003108. doi: 10.1371/journal.pgen.1003108 (PMC3521710; doi:10.1371/journal.pgen.1003108)
Supplement: Table S1 — Additional lifespan data. (DOCX) [file pgen.1003108.s006.docx]

**Table S1. Additional lifespan data.**

|  | **Temperature**^a^ | **Name** | **Strain** | **N**^b^ | **lifespan change** | **p-value**^c^ |
| --- | --- | --- | --- | --- | --- | --- |
| **Gain of Function** | 20^o^C | control | SD1507 | 140 | - | - |
|  |  | *egl-27::mCherry* | SD1601 | 140 | 29% | **6.4x10^-8^** |
|  |  | control | SD1507 | 262 | - | - |
|  |  | *egl-27::mCherry* | SD1601 | 334 | 8% | **3.1x10^-5^** |
|  |  | control | SD1507 | 72 | - | - |
|  |  | *egl-27::mCherry* | SD1601 | 29 | 60% | **0.0017** |
|  |  | control | SD1507 | 146 | - | - |
|  |  | *egl-27::GFP* | OP177 | 192 | 32% | **0.00016** |
|  | 15^o^C->15^o^C | control | SD1507 | 50 | - | - |
|  |  | *egl-27(we3) OE* | SD1622 | 119 | 11% | 0.152 |
|  |  | *egl-27(we3) OE* | SD1623 | 84 | 21% | 0.0733 |
|  |  | *egl-27(we3) OE* | SD1624 | 94 | 11% | 0.2 |
|  |  | control | SD1507 | 70 | - | - |
|  |  | *egl-27(we3) OE* | SD1622 | 84 | 92% | **4.9x10^-5^** |
|  |  | *egl-27(we3) OE* | SD1623 | 69 | 25% | **0.00085** |
|  |  | *egl-27(we3) OE* | SD1624 | 62 | 33% | 0.34 |
|  |  | control | SD1507 | 82 | - | - |
|  |  | *egl-27(we3) OE* | SD1622 | 127 | 6% | 0.145 |
|  |  | *egl-27(we3) OE* | SD1623 | 149 | 10% | 0.384 |
|  |  | *egl-27(we3) OE* | SD1624 | 135 | 13% | **0.00018** |
|  | 15^o^C->20^o^C | control | SD1507 | 61 | - | - |
|  |  | *egl-27(we3) OE* | SD1622 | 128 | 23% | **0.0051** |
|  |  | *egl-27(we3) OE* | SD1623 | 106 | 23% | **0.00072** |
|  |  | *egl-27(we3) OE* | SD1624 | 137 | 31% | **1.4x10^-5^** |
|  |  | control | SD1507 | 61 | - | - |
|  |  | *egl-27(we3) OE* | SD1622 | 95 | 8% | 0.636 |
|  |  | *egl-27(we3) OE* | SD1623 | 89 | 17% | 0.145 |
|  |  | *egl-27(we3) OE* | SD1624 | 65 | -8% | 0.767 |
|  |  | control | SD1507 | 104 | - | - |
|  |  | *egl-27(we3) OE* | SD1622 | 115 | 47% | **0.00065** |
|  |  | *egl-27(we3) OE* | SD1623 | ND^d^ | ND^d^ | ND^d^ |
|  |  | *egl-27(we3) OE* | SD1624 | 118 | 20% | 0.109 |
| **Loss of Function** | 20^o^C->15^o^C | WT | N2 | 95 | - | - |
|  |  | *egl-27(we3)* | JA1194 | 73 | -19% | **8.1x10^-7^** |
|  | 20^o^C | WT | N2 | 101 | - | - |
|  |  | *egl-27(we3)* | JA1194 | 100 | -8% | **9.9x10^-6^** |
|  |  | *daf-2(e1370)* | CB1370 | 86 | - | - |
|  |  | *daf-2(e1370); egl-27(we3)* | SD1625 | 72 | -59% | **0** |
|  |  | *daf-2(e1370)* | CB1370 | 96 | - | - |
|  |  | *daf-2(e1370); egl-27(we3)* | SD1625 | 52 | -62% | **0.0018** |
| **4mM Paraquat** | 20^o^C->15^o^C | WT | N2 | 160 | - | - |
|  |  | *egl-27(we3)* | JA1194 | 146 | -36% | **0** |
|  |  | *daf-2(e1370)* | CB1370 | 191 | - | - |
|  |  | *daf-2(e1370); egl-27(we3)* | SD1625 | 205 | -70% | 0.0529 |
|  |  | control | SD1507 | ND^d^ | ND^d^ | ND^d^ |
|  |  | *egl-27::GFP* | OP177 | 190 | -7%* | **0.0014** |
| **Heat** | 20^o^C | N2 | N2 | 105 | - | - |
|  |  | *egl-27(we3)* | JA1194 | 109 | -75% | **1.7x10^-8^** |
|  |  | *daf-2(e1370)* | CB1370 | 58 | - | - |
|  |  | *daf-2(e1370); daf-16(mu86)* | CF1588 | 117 | -79% | **3.9x10^-14^** |
|  |  | *daf-2(e1370); egl-27(we3)* | SD1625 | 85 | -16% | 0.63 |
|  |  | control | SD1507 | ND^d^ | ND^d^ | ND^d^ |
|  |  | *egl-27::GFP* | OP177 | 81 | 56%* | **6.1x10^-10^** |

^a^ When shift is indicated, worms are hatched at first temperature and shifted to second temperature at day 2 of adulthood.

^b^ Number of worms

^c^ Log-rank p-value, bold values indicate p<0.05

^d^ Experiment not done

* Lifespan change and p-value in comparison to N2 rather than co-injection control because control lifespan was not done.
