# Supplementary material for: The GATA Transcription Factor egl-27 Delays Aging by Promoting Stress Resistance in Caenorhabditis elegans
Source: PLoS Genet. 2012 Dec 13;8(12):e1003108. doi: 10.1371/journal.pgen.1003108 (PMC3521710; doi:10.1371/journal.pgen.1003108)
Supplement: Table S4 — Top GO categories enriched in EGL-27 targets. (DOCX) [file pgen.1003108.s009.docx]

**Table S4. Top GO categories enriched in EGL-27 targets**

| **All EGL-27 targets** | | |
| --- | --- | --- |
| **GO Category** | **q-value^a^** | **Enrichment** |
| structural constituent of cuticle | 2.9E-05 | 4.8 |
| aromatic compound catabolic process | 6.5E-03 | 31 |
| **Age-dependent EGL-27 targets** | | |
| **GO Category** | **q-value^a^** | **Enrichment** |
| structural constituent of cuticle | 5.83E-13 | 20.32 |
| structural molecule activity | 1.19E-06 | 7.12 |
| aromatic amino acid family metabolic process | 7.69E-04 | 68.17 |
| aromatic amino acid family catabolic process | 6.85E-04 | 153.39 |
| organic acid metabolic process | 5.74E-04 | 8.77 |
| cellular ketone metabolic process | 5.03E-04 | 8.48 |
| aromatic compound catabolic process | 4.88E-04 | 122.71 |
| cellular aromatic compound metabolic process | 1.16E-03 | 14.78 |
| small molecule catabolic process | 1.45E-03 | 20.45 |
| oxidation-reduction process | 5.10E-03 | 4.73 |

GO analysis performed using GOrilla [1,2]

^a^ Hypergeometric p-value corrected for multiple hypothesis testing using the Benjamini and Hochberg method.

1. Eden E, Lipson D, Yogev S, Yakhini Z (2007) Discovering motifs in ranked lists of DNA sequences. PLoS Comput Biol 3: e39. doi:10.1371/journal.pcbi.0030039.

2. Eden E, Navon R, Steinfeld I, Lipson D, Yakhini Z (2009) GOrilla: a tool for discovery and visualization of enriched GO terms in ranked gene lists. BMC Bioinformatics 10: 48. doi:10.1186/1471-2105-10-48.
