# Supplementary material for: The GATA Transcription Factor egl-27 Delays Aging by Promoting Stress Resistance in Caenorhabditis elegans
Source: PLoS Genet. 2012 Dec 13;8(12):e1003108. doi: 10.1371/journal.pgen.1003108 (PMC3521710; doi:10.1371/journal.pgen.1003108)
Supplement: Table S5 — Description of all stress microarray datasets used. (DOCX) [file pgen.1003108.s010.docx]

**Table S5. Description of all stress microarray datasets used.**

| **Stress** | **Description** | **Publication** | **# filtered genes** |
| --- | --- | --- | --- |
| ***E. carotovora*** | worms fed *E. carotovara* for 24h | Wong *et al.* (2007). *Genome Biology* [72] | 959 |
| ***E. faecalis*** | worms fed E. *faecalis* for 24h | Wong *et al.* (2007). *Genome Biology* [72] | 1161 |
| ***P. aeruginosa*** | worms fed PA14 for 4h or 8h | Troemel *et al.* (2006). *PLoS Gen* [73] | 634 |
| ***P. luminescens*** | worms fed *P. luminescens* for 24h | Wong *et al.* (2007). *Genome Biology* [72] | 1146 |
| ***S. aureus*** | worms fed *S. aureus* for 8h | Irazoqui *et al.* (2010). *PLoS Pathogens* [74] | 386 |
| ***S. marcesens*** | worms fed *S. marcesens* for 24h | Wong *et al.* (2007). *Genome Biology* [72] | 1120 |
| **Cry5b** | worms fed *E. coli* expressing pore-forming Crystal toxin Cry5b for 3h | Huffman et al. (2004). PNAS [75] | 1012 |
| **Cadmium** | worms grown on plates supplemented with cadmium | Huffman *et al.* (2004). *PNAS* [75] | 992 |
| **Ethanol** | worms cultured in 7% ethanol for 15 minutes, 30 minutes, and 6h | Kwon *et al.* (2004). *Genomics* [76] | 219 |
| **Silver** | worms cultured in 0.1mg/L silver nanoparticles for 24h | Roh *et al.* (2009). *Environ. Sci. Technol.* [77] | 1519 |
| **Heat** | 30^o^C for 24h | Mongkoldhumrongkul *et al.* (2010). *J.R. Soc Interface* [52] | 614 |
| **Hypo-osmotic** | worms grown on plates with 200nM NaCl for 15 minutes, 1h, 6h, and 1 generation | Rohlfing *et al.* (2010). *PLoS ONE* [49] | 313 |
| **Oxidative** | 99% O_2_ at 40 psi for 6h | Park *et al.* (2009). *Aging Cell* [55] | 1243 |
| **Gamma Radiation** | worms exposed to 120Gy gamma; harvested 2h or 6h post-treatment | Greiss *et al.* (2008). *BMC Genomics* [78] | 1946 |
| **X-Ray** | worms exposed to 120Gy X-ray; harvested 2h post-treatment | Greiss *et al.* (2008). *BMC Genomics* [78] | 607 |
| **Starvation** | starvation induced L1 arrest | Baugh *et al.* (2009). *Science* [79] | 3479 |
