# Supplementary material for: The GATA Transcription Factor egl-27 Delays Aging by Promoting Stress Resistance in Caenorhabditis elegans
Source: PLoS Genet. 2012 Dec 13;8(12):e1003108. doi: 10.1371/journal.pgen.1003108 (PMC3521710; doi:10.1371/journal.pgen.1003108)
Supplement: Table S6 — Strains. (DOCX) [file pgen.1003108.s011.docx]

**Table S6. Strains.**

| **Strain** | **Genotype** | **Figures** | **Reference / Description** |
| --- | --- | --- | --- |
| **OP177** | *unc-119(ed3)III; wgIs177[egl-27::GFP::3xFLAG; unc-119(+)]* | 1, 2, 5, S5, Tables 1, S1-S4 | Gerstein *et al.*, 2010; Niu *et al.* 2011 [44,65] |
| **SD1507** | *unc-119(ed3)III; Ex(Psod-3::mCherry; unc-119(+)]* | 1, 2, S1, S5, Table S1 | Sagi and Kim, 2012 [54] |
| **JA1194** | *egl-27(we3)II* | 1, 2, 6, Table S1 | Solari *et al.*, 1999 [30] |
| **CB1370** | *daf-2(e1370)III* | 1, 2, S1, S2, Table S1 | Kenyon *et al.* 1993; Larsen *et al.*, 1995 [34,80] |
| **SD1625** | *daf-2(e1370)III; egl-27(we3)II* | 1, 2, S1, Table S1 | This study |
| **CF1588** | *daf-2(e1370); daf-16(mu86)I; muls84[pAD76(sod-3::GFP)]* | 1, 2, Table S1 | Libina *et al.*, 2003 [36] |
| **SD1751** | *egl-27(we3)II; wgIs177[Pegl-27::GFP::3xFLAG; unc-119(+)]* | 1 | This study |
| **SD1601** | *unc-119(ed3)III; gaIs277[egl-27::3x FLAG::cherry; unc-119(+)]* | 1, S1, Table S1 | This study; Biolistic bombardment |
| **SD1584** | *unc-119(ed3)III; stIs10161[Pegl-27::mCherry; unc-119(+)]; ccIs4251[Pmyo-3::GFP; unc-119(+)]* | 3, 4, S2, S3 | Liu *et al.*, 2009 [81] |
| **SD1776** | *daf-2(e1370)III; stIs10161[Pegl-27::mCherry; unc-119(+)]; ccIs4251[Pmyo-3::GFP; unc-119(+)]* | 3, S2 | This study |
| **SD1778** | *daf-2(e1370)III; daf-16(mu86)I; stIs10161[Pegl-27::mCherry; unc-119(+)]* | 3, S2 | This study |
| **SD1777** | *daf-2(e1370)III; elt-3(vp1)X; stIs10161[Pegl-27::mCherry; unc-119(+)]* | 3, S2 | This study |
| **SD1809** | *elt-3(vp1)X; stIs10161[Pegl-27::mCherry; unc-119(+)]; ccIs4251[Pmyo-3::GFP; unc-119(+)]* | 3, S2 | This study |
| **SD1862** | *egl-27(we3)II; stIs10161[Pegl-27::mCherry; unc-119(+)]; ccIs4251[myo-3::GFP; unc-119(+)]* | 3, S2 | This study |
| **SD1746** | *egl-27(we3)II; muIs84[pAD76(sod-3::GFP)]* | 3 | This study |
| **SD1743** | *daf-2(e1370)III; egl-27(we3)II; muIs84[pAD76(sod-3::GFP)]* | 3 | This study |
| **CF1553** | *muIs84[pAD76(sod-3::GFP)]* | 3, S2 | Libina *et al.*, 2003; Sanchez-Blanco and Kim, 2011 [36,37] |
| **CF1580** | *daf-2(e1370)III; muIs84[pAD76(sod-3::GFP)]* | 3, S2 | Libina *et al.*, 2003; Sanchez-Blanco and Kim, 2011 [36,37] |
| **SD1622** | *unc-119(ed3)III; Ex[egl-27; Psod-3::H1B::mCherry; unc-119(+)]* | S1, Table S1 | This study; injection of 20ng/uL *egl-27(we3)*, 80ng/uL *Psod-3::H1B::mCherry; unc-119(+)* |
| **SD1623** | *unc-119(ed3)III; Ex[egl-27; Psod-3::H1B::mCherry; unc-119(+)]* | S1, Table S1 | This study; injection of 20ng/uL *egl-27(we3)*, 80ng/uL *Psod-3::H1B::mCherry; unc-119(+)* |
| **SD1624** | *unc-119(ed3)III; Ex[egl-27; Psod-3::H1B::mCherry; unc-119(+)]* | S1, Table S1 | This study; injection of 20ng/uL *egl-27(we3)*, 80ng/uL *Psod-3::H1B::mCherry; unc-119(+)* |
