# Supplementary material for: The GATA Transcription Factor egl-27 Delays Aging by Promoting Stress Resistance in Caenorhabditis elegans
Source: PLoS Genet. 2012 Dec 13;8(12):e1003108. doi: 10.1371/journal.pgen.1003108 (PMC3521710; doi:10.1371/journal.pgen.1003108)
Supplement: Table S7 — Primers. (DOCX) [file pgen.1003108.s012.docx]

**Table S7. Primers.**

| **qPCR primers** | | |
| --- | --- | --- |
| **Name** | **Sequence** | **TM** |
| ***act-1* F** | CACGGTATCGTCACCAACTG | 60^o^C |
| ***act-1* R** | GCTTCAGTGAGGAGGACTGG | 60^o^C |
| ***egl-27* F** | CCATCAGGAAGAGCGTGTCA | 60^o^C |
| ***egl-27* R** | CACGCTGGAACTTGAGATGG | 60^o^C |
| ***grd-3* F** | ACACCTTGGCCATCTACGAG | 60^o^C |
| ***grd-3* R** | AGGTGCTTGGAAACTGATGG | 60^o^C |
| ***T14B1.1* F** | AACCGGGAACAGAACGGAGC | 60^o^C |
| ***T14B1.1* R** | TCCTGGTCGGCTTCATGATCAAA | 60^o^C |
| ***Y37A1B.5* F** | TCAGCCGAGTTGATGTGCCA | 60^o^C |
| ***Y37A1B.5* R** | ACGGTCAGAGTTCAGGCAAG | 60^o^C |
| ***lpr-3* F** | AAGCTGCCGGAGTCAAGACC | 60^o^C |
| ***lpr-3* R** | TGGTTGCTCAGCTGGAAGTG | 60^o^C |
| **Genotyping primers** | | |
| **Name** | **Sequence** | **TM** |
| ***daf-2(e1370)* inner F** | CACCTCATCATTACTCAAACCAATATAGGG | 58^o^C |
| ***daf-2(e1370)* inner R** | TATGAAATGGTTACACTCGGTGCTCCGT | 58^o^C |
| ***daf-2(e1370)* outer F** | GGTGAGTATCTCCAGCACATTTTCATCA | 58^o^C |
| ***daf-2(e1370)* outer R** | CGGAATGGCTCGTGATCTATTCTATCAT | 58^o^C |
| ***egl-27(we3)* inner F** | AACAAATCCACCAGCAGCAAGCTCCGT | 63^o^C |
| ***egl-27(we3)* inner R** | TTGAAGTTGTTGCAGATGTTGAGCATTGTG | 63^o^C |
| ***egl-27(we3)* outer F** | TCCTCGGTGTGCACGAACCGATTTAATT | 63^o^C |
| ***egl-27(we3)* outer R** | AAGAGCCATTTGATGTTGGAATGCTGCA | 63^o^C |
| ***daf-16(mu86) F*** | CCCACATTCGTGTGGGTTTTCTAGTCG | 50^o^C |
| ***daf-16(mu86) int R*** | CGTTATCAAATGCTCCTTGCATTGAATC | 50^o^C |
| ***daf-16(mu86) out R*** | GCGTCAGTTCCGATCTGATATGAAC | 50^o^C |
| ***elt-3(vp1) F*** | CAAACTTCGCAACATTCCAACCAGC | 55^o^C |
| ***elt-3(vp1) int R*** | CAGTGCTTGTTATGTCTTTCTCGG | 55^o^C |
| ***elt-3(vp1) out R*** | CCATCTTTTCTAAGAGACAGTGGACG | 55^o^C |
